# METTL17 is an Fe-S cluster checkpoint for mitochondrial translation

**DOI:** 10.1016/j.molcel.2023.12.016

**Published:** 2024-01-09

**Authors:** Tslil Ast, Yuzuru Itoh, Shayan Sadre, Jason G. McCoy, Gil Namkoong, Jordan C. Wengrod, Ivan Chicherin, Pallavi R. Joshi, Piotr Kamenski, Daniel L. M. Suess, Alexey Amunts, Vamsi K. Mootha

**Affiliations:** 1- Broad Institute, Cambridge, MA 02142, USA; 2- Howard Hughes Medical Institute, Massachusetts General Hospital, Boston, MA 02114, USA; 3- Department of Molecular Biology, Massachusetts General Hospital, Boston, MA 02114, USA; 4- Department of Systems Biology, Harvard Medical School, Boston, MA 02115, USA; 5- Science for Life Laboratory, Department of Biochemistry and Biophysics, Stockholm University, Solna, Sweden; 6- Department of Chemistry, Massachusetts Institute of Technology, Cambridge, MA 02139, USA; 7- Department of Biology, M.V.Lomonosov Moscow State University, Moscow, 119234, Russia; 8- Corresponding author

## Abstract

Friedreich’s ataxia (FA) is a debilitating, multisystemic disease caused by a depletion of frataxin (FXN), a mitochondrial iron-sulfur (Fe-S) cluster biogenesis factor. To understand the cellular pathogenesis of FA, we performed quantitative proteomics in FXN-deficient human cells. Nearly every annotated Fe-S cluster-containing protein was depleted, indicating that as a rule, cluster binding confers stability to Fe-S proteins. We also observed depletion of a small mitoribosomal assembly factor METTL17 and evidence of impaired mitochondrial translation. Using comparative sequence analysis, mutagenesis, biochemistry and cryoelectron microscopy we show that METTL17 binds to the mitoribosomal small subunit during late assembly and harbors a previously unrecognized [Fe_4_S_4_]^2+^ cluster required for its stability. METTL17 overexpression rescued the mitochondrial translation and bioenergetic defects, but not the cellular growth, of FXN-depleted cells. These findings suggest that METTL17 acts as an Fe-S cluster checkpoint: promoting translation of Fe-S cluster rich OXPHOS proteins only when Fe-S cofactors are replete.

## eTOC Blurb

Ast et al. examined the cellular consequences of frataxin deficiency, modeling Friedreich’s ataxia. They discovered a decrease in METTL17, an essential mitoribosome assembly factor. They show that METTL17 binds a Fe-S cluster, which is critical for its stability. Overexpression of METTL17 rectifies the bioenergetic deficits of Friedreich’s ataxia.

## Graphical Abstract

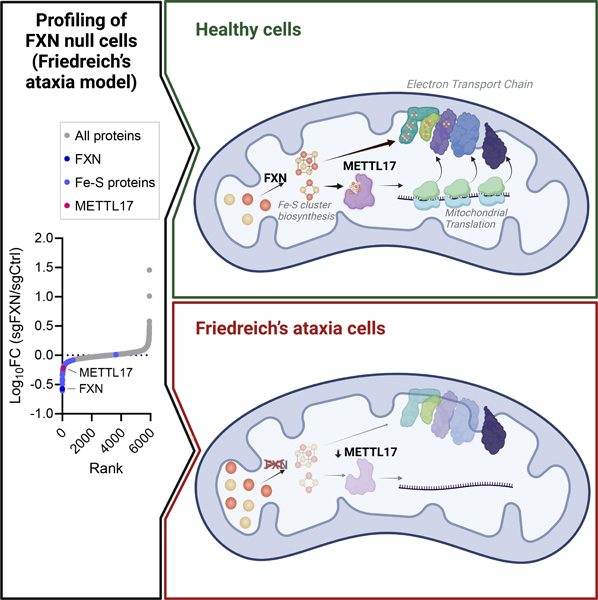

## Introduction

Friedreich’s ataxia (FA) is a progressive neurological disorder impacting 1 in 50,000 people^1–3^. While the primary feature of FA is ataxia, this disease is in fact multisystemic. Patients can also develop diabetes, scoliosis, hearing and vision loss, as well as cardiomyopathy, the latter being a leading cause of premature mortality at a median age of 37.5 years^4,5^. FA is caused by a recessive depletion of a nuclear-encoded mitochondrial protein frataxin (FXN)^6^, that functions as an allosteric activator of iron-sulfur (Fe-S) cluster biosynthesis^7–10^. Tissue and cell samples from FA patients contain 5–30% residual FXN levels^11,12^. This depletion is most often due to the expansion of a naturally occurring GAA track found within the first intron of the gene^6,13–15^ which triggers loss of FXN expression^16–18^. FA is the most common monogenic mitochondrial disease and the most common inherited ataxia. Yet we still lack truly effective therapies for FA, and the mainstay of therapy focuses on symptomatic management.

Fe-S clusters are ancient and universal redox cofactors^19–22^. In humans, there are ~60 known Fe-S cluster binding proteins^23,24^ that operate throughout the mitochondrion, cytosol and nucleus. For all these subcellular compartments, cluster biosynthesis is initiated in the mitochondria where FXN accelerates Fe-S cluster formation^8–10^. These versatile cofactors play a number of different roles; while the most widely appreciated one is electron transfer, they also participate in enzyme catalysis, sulfur donation, and redox sensing^7,21,24^. In addition, there is evidence that in some cases Fe-S clusters contribute to the structural stability of the proteins into which they are integrated. Once bound to their co-factor, Fe-S cluster binding proteins take part in diverse cellular pathways such as DNA replication and repair, nucleotide biosynthesis and energy metabolism^7,25^. Indeed, in FA it has been well documented in patient samples as well as several disease models that there is a deficit in mitochondrial oxidative phosphorylation (OXPHOS) leading to bioenergetic defects^26–30^. It has been suggested that one reason for the neuronal and cardiac deficits observed in FA might be the high-energy demand of these tissues^31–33^.

To systematically understand the cellular consequences of FXN loss, we performed both proteomic and genetic interaction mapping on the background of FXN deficiency. Unexpectedly, we find that a consequence of FXN loss is a significant impairment in mitochondrial protein synthesis. We determine that METTL17 – a conserved mitoribosome assembly factor^34^ – harbors a previously unrecognized Fe-S cluster that stabilizes its binding to the mitoribosomal small subunit. As a result, in FXN deficient cells, METTL17 activity is diminished. The data suggest that METTL17 loss is a mitochondrial repercussion of FXN deficiency and contributes to an intra-organelle translational defect.

## Results

### Loss of FXN leads to a widespread depletion of Fe-S cluster containing proteins

**Fig 1:**
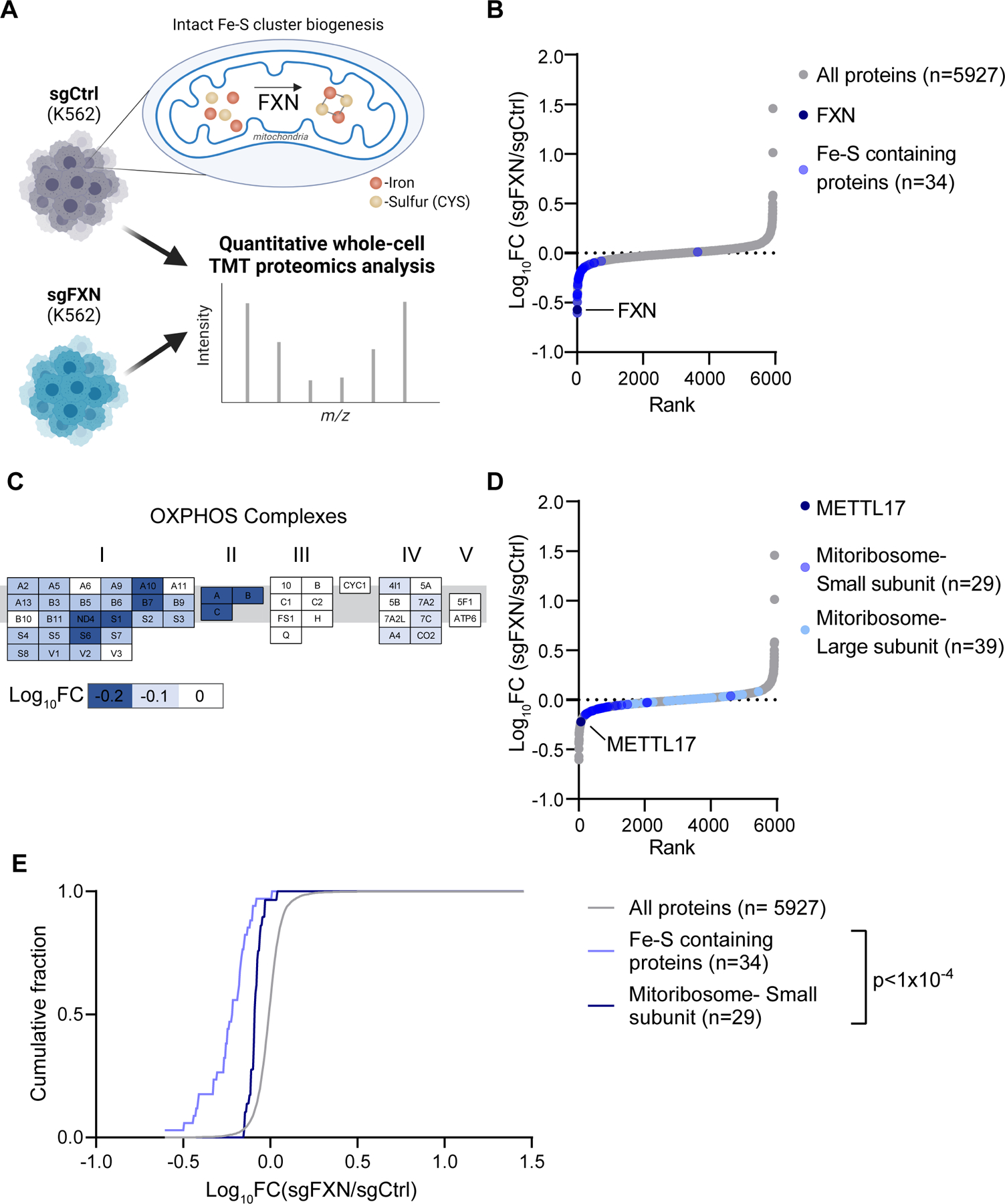
Proteomic analysis of FXN edited cells reveals a marked depletion of known Fe-S cluster containing proteins and reduction of small mitoribosome subunits. A. Quantitative whole cell proteomic analysis was carried out on K562 cells edited with control or FXN targeted guides in duplicate, depleting for this allosteric regulator of Fe-S cluster biosynthesis. B. Waterfall plot of protein fold change in FXN/Control edited cells, highlighting FXN and validated human Fe-S cluster containing proteins. C. OXPHOS proteins are organized by complex with blue indicating proteins that are depleted in FXN edited cells. Genes are ordered alphabetically within complex using complex-specific prefixes (NDUF, SDH, UQCR, COX, ATP5) (e.g. A2 in CI refers to NDUFA2 whereas A in CII refers to SDHA). D. Waterfall plot of protein fold change in FXN/Control edited cells, highlighting proteins in the small and large mitoribosome subunit, as well as the small subunit assembly factor, METTL17. E. Cumulative distribution of Fe-S containing proteins and proteins of the small mitoribosome subunit versus all identified proteins in FXN/Control edited cells. ****=p < 0.0001. Two sample Kolmogorov-Smirnov test. See also Fig S1 and Table S2.

We sought to determine the cellular consequences of loss of FXN, an allosteric activator of Fe-S cluster biosynthesis found in the mitochondria. We performed quantitative, whole-proteome profiling of human K562 cells following polyclonal CRISPR-based disruption of *FXN* versus *OR11A1,* a non-expressed gene that serves as an editing control, in duplicate (Fig 1A). In this analysis (Table S2), FXN itself scored as the 2^nd^ most depleted protein (Fig 1B), while its partner ISC machinery (ISCU, NFS1, LYRM4) remained unchanged (Fig S1A). On the upgoing side, IRP2, an iron regulatory protein known to be stabilized in FXN deficiency, was among the top 100 upregulated proteins. In addition, one of the top upgoing proteins was an apolipoprotein B receptor, APOBR, consistent with recent work showing a striking change in cellular lipid metabolism following the loss of Fe-S cluster biosynthesis^35–38^. Notably, of the 34 known Fe-S cluster containing proteins detected in our proteomics analysis, 33 were downregulated (Fig 1B). The only unaffected Fe-S cluster containing protein was NARF, a nuclear protein that is a component of a prelamin-A endoprotease complex^39^. This analysis revealed that depletion of FXN leads to a near universal diminution in Fe-S cluster binding proteins. While many Fe-S cluster containing proteins are known to depend on their cofactor for stability^7,21,24^, the ubiquity of this phenomenon has not been systematically reported. It is likely that this pervasive depletion takes place at the post-transcriptional level, as these proteins function in and are regulated by distinct pathways. The ubiquity of this depletion suggests that proper incorporation of an Fe-S cluster is essential for the structural integrity of virtually all human Fe-S cluster binding proteins.

### FXN loss results in a decrease of small mitoribosome subunits and its assembly factor METTL17

We next asked at an organelle-wide level what pathways are most altered following FXN depletion. We systematically considered effects to the mitochondrial proteome, analyzing all 149 annotated pathways (called MitoPathways) described in MitoCarta 3.0, a freely available inventory of high-confidence mitochondrial localized mammalian proteins^40^. We found that apart from Fe-S containing proteins, the levels of respiratory chain Complexes I, II and IV subunits were reduced (Fig 1C and Fig S1B). Indeed, Complexes I, II and III contain Fe-S clusters, and moreover, biopsy material from FA models will often exhibit moderate defects in the Complexes I and II^29,30^. Of note, our analysis did not reveal a widespread decrease in all mitochondrial-localized proteins (Fig S1C), indicating that the overall mitochondrial content is preserved despite loss of FXN.

We were intrigued to see that cells edited for *FXN* also displayed reduced levels of the mitochondrial ribosome, specifically proteins that make up the small (SSU), but not large (LSU) subunit of the mitochondrial ribosome (Fig 1D and Fig S1B). The mitoribosome is essential for the intra-organellar translation of the 13 mtDNA encoded OXPHOS subunits in humans^41–43^, directly supporting the formation of Complexes I, III, IV and V. Indeed, without intra-organellar translation, mitochondrial respiration cannot occur. Further examination of other proteins involved in mtDNA expression, i.e., mtDNA maintenance and mtRNA metabolism, revealed no such similar reduction in *FXN* edited cells (Fig S1D). Intriguingly, one of the top depleted proteins in the FXN-edited cells was METTL17 (Fig 1D), a seven-beta-strand methyltransferase which has recently been suggested to function as a SSU assembly factor^34^ but has no known association with Fe-S cluster biosynthesis.

Collectively these proteomics analyses revealed a very consistent decrease in the abundance of Fe-S cluster containing proteins as well as components of the small mitochondrial subunit. Both of these pathways were significantly left-shifted (Kolmogorov-Smirnov D-statistic P<0.0001) relative to background (Fig 1E). Mitochondrial proteins, as a whole, were not left shifted. These findings indicated a profound, yet poorly elucidated, connection between Fe-S cluster levels and the mitochondrial ribosome, which we sought to further explore.

### Mitochondrial translation is attenuated in the absence of FXN

**Fig 2:**
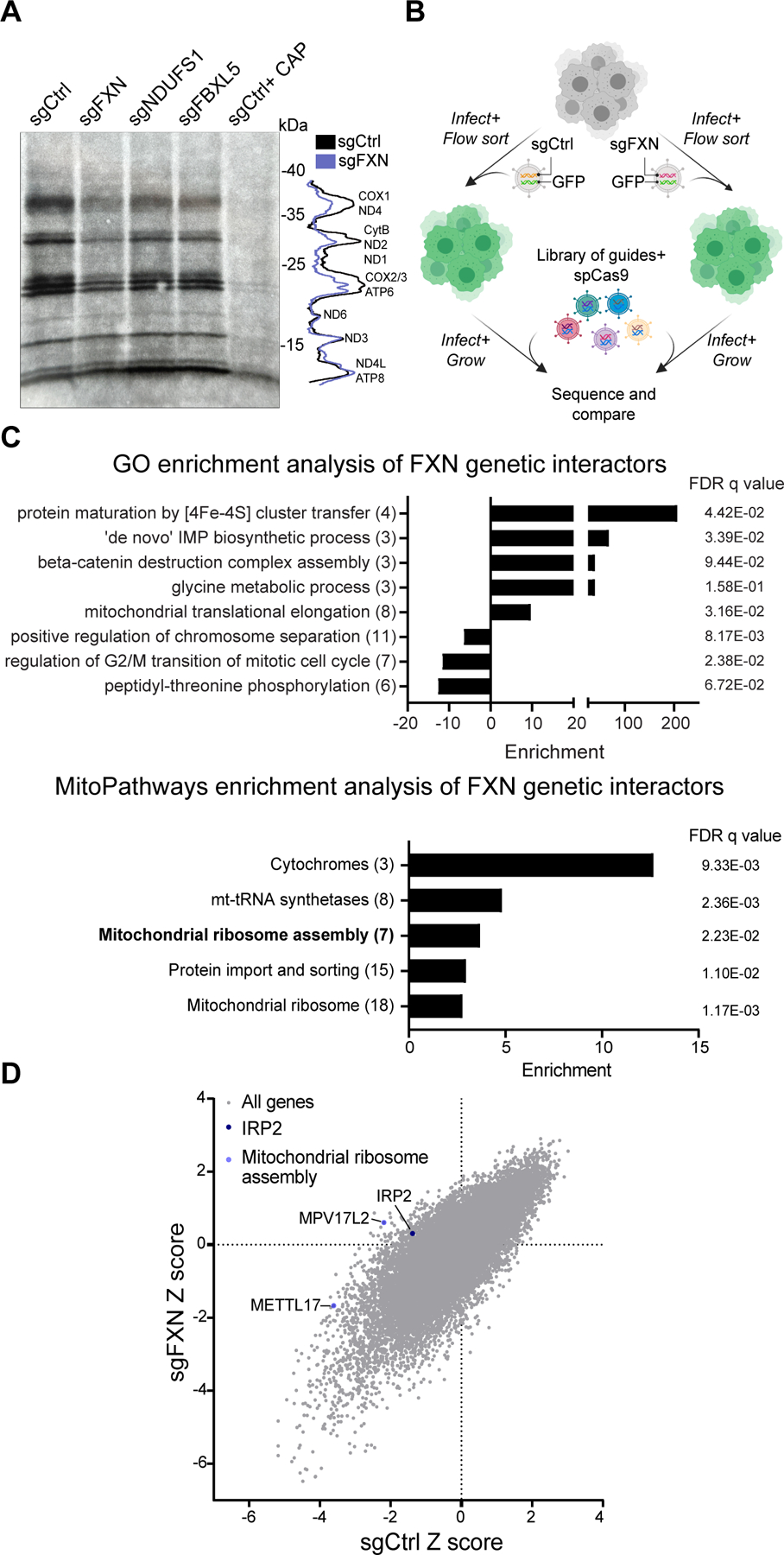
Mitochondrial translation is attenuated in the absence of FXN. A. Left-Mitochondrial translation, as assessed by autoradiography after ^35^S-methionine/cysteine labeling, of cells expressing sgRNAs targeting *FXN*, *NDUFS1* or *FBXL5*. All cells were treated with 200μg/mL emetine, and control cells in the last lane were also treated with 50μg/μL chloramphenicol. Right-Quantification of the band intensities in control vs. *FXN* edited cells. B. Schematic overview of the genome-wide CRISPR genetic interaction screens carried out in K562 cells. Cells were either infected with guides against FXN or a control locus before introduction of the library. Following expansion, cells were sequenced to assess the relative abundance of guides in the FXN depleted vs. control background. C. GO (top) and MitoCarta 3.0 (bottom) enrichment analysis of genetic interactors identified in for FXN depleted cells. In parentheses are the number of genes enriched in the screen for each term. D. Scatterplot of Z scores showing knockouts growth in sgCtrl vs. sgFXN backgrounds. The positive control (IRP2) and the mitochondrial ribosome assembly genes (METTL17 and MPV17L2) are highlighted. See also Fig S2 and Table S3.

We next directly tested whether FXN depletion results in a defect in intra-mitochondrial translation. To this end, we assessed the rates of protein synthesis in the mitochondria in K562 cells edited for *FXN*, *NDUFS1* or *FBXL5* (Fig 2A and Fig S2A–B). The latter two genes would indicate whether any changes we observe in *FXN* edited cells are triggered by known secondary effects, as *NDUFS1* encodes for a subunit of respiratory chain Complex I^44–46^ while *FBXL5* is an Fe-S cluster binding protein that acts as a negative regulator of the iron-starvation response^47–49^. While *FXN* deficient cells had dampened intra-mitochondrial protein synthesis, we did not observe a comparable effect in *NDUFS1* or *FBXL5* deficient cells. This translation defect was not a secondary consequence of a defect in mtDNA homeostasis or expression, as mtDNA copy number and mtRNA levels were intact upon loss of FXN (Fig S2C–D). Furthermore, mitochondrial translation was also attenuated when other components of the Fe-S cluster biosynthesis machinery, such as the cluster scaffold, *ISCU,* or the cysteine desulfurase, *NFS1,* were edited (Fig S2E–F).

We wondered which of the many genetic pathways downstream of *FXN* deficiency contribute to the reduced fitness observed in these cells^50^. We performed a genome-wide CRISPR screen (Fig. 2B) on a FXN edited background to highlight pathways that become conditionally essential (synthetic sick) vs. better-than-expected or epistatic (buffering)^51^. The screen, performed in duplicate, worked from a technical perspective (Fig S2G); we recovered positive genes / pathways (e.g., previously established buffering genetic interaction with Iron Regulatory Protein 2, *IRP2,* in 21% O_2_ ; Fig 2D)^50^ and Fe-S cluster dependent pathways (e.g., *de novo* IMP biosynthesis caused by a loss of the Fe-S binding protein PPAT; Fig 2C) in the screen. Intriguingly, enrichment analysis using either GO terms or MitoPathways (from MitoCarta 3.0) highlighted mitochondrial translation as one of the key cellular pathways that was buffered in *FXN* edited cells (Fig 2C). Specifically, MitoPathways enrichment highlighted that a key deficit in *FXN* edited cells is mitochondrial ribosome assembly, with loss of both the mitoribosome assembly factors MPV17L2 and METTL17 scoring as buffering interactions in this screen (Fig 2D and Fig S2H–I). Likewise, genes encoding for mitoribosome subunits also were enriched in this screen, showing significant buffering interactions with loss of FXN (Fig 2C). These genetic interactions pointed to METTL17 as worthy of additional investigation.

### METTL17 is essential to maintain robust mitochondrial translation and is post-transcriptionally depleted in the absence of FXN

METTL17 is a highly conserved mitochondrial matrix protein that has recently been linked to mitochondrial translation^52^. Specifically, METTL17 belongs to the methyltransferase-like family^53^ and has been suggested to take part in the assembly of the mitoribosomal small subunit ^34^. However, with respect to the mitoribosome, its methylation target remains controversial ^54–56^ and so the contribution of METTL17 to mitoribosome maturation remains unresolved.

**Fig 3:**
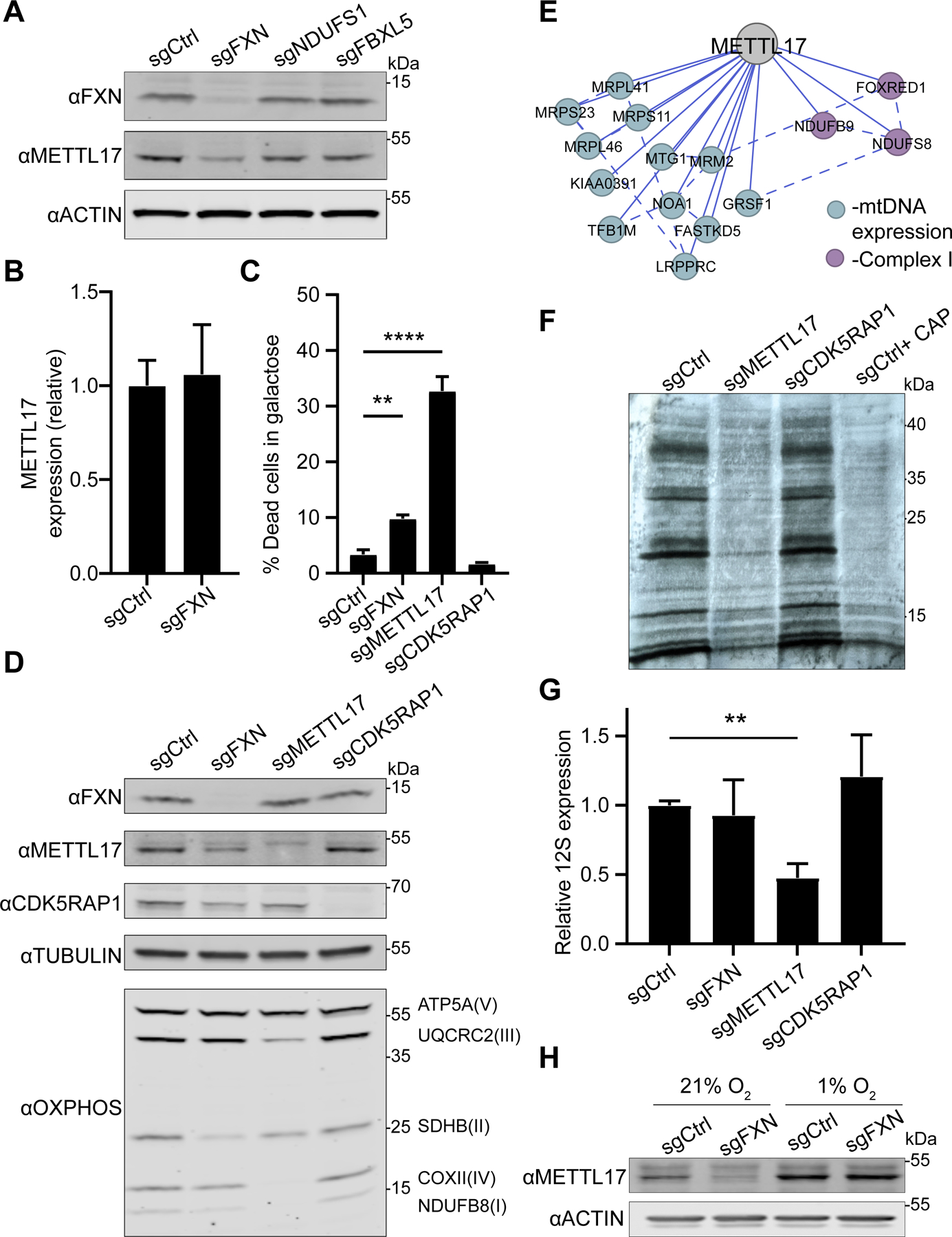
METTL17 is depleted in the absence of FXN and is essential for robust mitochondrial translation. A. Immunoblot for FXN, METTL17 and the loading control actin in K562 cells edited with control, FXN, NDUFS1 and FBXL5 guides. B. qPCR for METTL17 expression levels in sgCtrl and sgFXN cells (n=8 for all samples) C. Cells edited for control, FXN, METTL17 and CDK5RAP1 genes were grown for 24h in galactose media, and viability was assessed for each background (n=4 for all samples) . D. Immunoblot for FXN, METTL17, CDK5RAP1, select OXPHOS subunits and the loading control tubulin in cells edited with control, FXN, METTL17 and CDK5RAP1 guides. E. Correlation analysis of gene dependencies sourced from DepMap. Presented is the gene network that correlates with METTL17 deletion using FIREWORKS^90^. Solid and dashed lines represent primary and secondary correlations, respectively. F. Mitochondrial translation, as assessed by autoradiography after ^35^S-methionine/cysteine labeling, of cells expressing sgRNAs targeting METTL17 or CDK5RAP1. All cells were treated with 200μg/mL emetine, and control cells in the last lane were also treated with 50μg/μL chloramphenicol. G. qPCR analysis of 12S levels in cells edited with control, FXN, METTL17 or CDK5RAP1 guides (n=8 for all samples). H. Immunoblot for METTL17 and the loading control actin in cells edited with control or FXN guides. Following editing, cells were grown in 21% or 1% oxygen. All bar plots show mean ± SD. **=p < 0.01, ****=p < 0.0001. Two-way ANOVA with Bonferroni’s post-test. See also Fig S3.

First, we validated our proteomics result that METTL17 is indeed depleted in *FXN* edited cells (Fig 3A, Fig S3A–B). Moreover, this depletion could not be phenocopied upon loss of *NDUFS1* or *FBXL5*, indicating that it was likely not due to a secondary consequence of OXPHOS deficiency or iron starvation signaling. Importantly, *METTL17* mRNA levels were preserved in *FXN* edited cells (Fig 3B), indicating that METTL17 protein is lost at the post-transcriptional level in the absence of FXN.

Next, we sought to determine the extent to which METTL17 depletion attenuates mitochondrial translation and OXPHOS. *METTL17* edited cells undergo extensive cell death when grown on galactose (Fig 3C and Fig S3C), a sugar source that forces cells to rely exclusively on OXPHOS for ATP production^52^. No significant galactose-induced death was observed in the absence of CDK5RAP1, a *bona-fide* Fe-S cluster dependent tRNA methylthiotransferase dually localized to the mitochondria and nucleus^57^. Furthermore, *METTL17* edited cells displayed combined respiratory chain deficiencies (Fig 3D), in contrast to *CDK5RAP1* depleted cells. METTL17 likely plays a pervasive and crucial role in the maturation of the SSU, consistent with its Cancer Dependency Map profile (Fig 3E), as well as its extensive physical interaction with SSU components identified in BioPlex (Fig S3D)^58^. In line with these unbiased methods, we observed near complete ablation of mitochondrial translation and depletion of the 12S (the SSU rRNA) in the absence of METTL17 (Fig 3F–G and Fig S3E). We noted that *METTL17* edited cells appear to have an even more extreme phenotype than *FXN* edited cells in many of these assays. This may be due to the kinetics of METTL17 depletion in *METTL17* versus *FXN* edited cells, the latter experiencing the METTL17 deficiency as a secondary effect which takes time to build up. Finally, we tested whether hypoxia could restore METTL17 levels following loss of *FXN*, as we have previously seen that Fe-S cluster synthesis is maintained under these conditions regardless of the absence of *FXN*^50^. Indeed, culturing *FXN* edited cells in 1% O_2_ was sufficient to revert METTL17 levels back to control conditions (Fig 3H), consistent with it being a direct outcome of FXN deficiency. Collectively, these experiments confirm that METTL17 is intimately linked to SSU functionality in a way that is dependent on intact FXN levels.

### METTL17 contains a putative Fe-S cluster binding motif essential for its function

**Fig 4:**
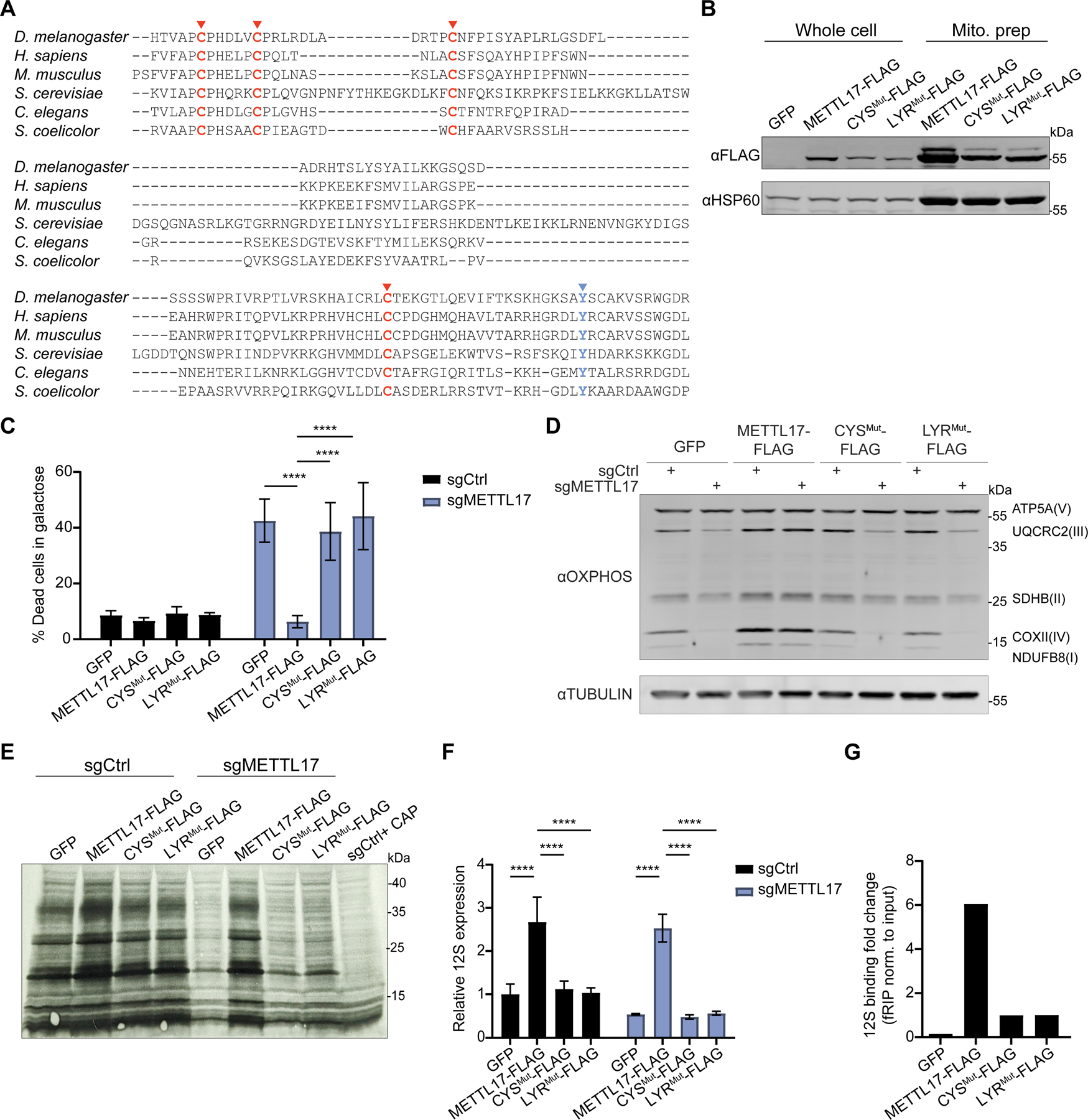
METTL17 harbors conserved motifs for Fe-S binding that are crucial for its functionality. A. Multiple sequence alignment for METTL17 homologues, highlighting two motifs associated with Fe-S cluster binding; 4 cysteine metal binding pocket (red) and a LYR handoff motif (blue). B. Immunoblot from whole cell and mitoprep extracts of cells expressing GFP, METTL17-FLAG, CYS^Mut^-FLAG or LYR^Mut^-FLAG constructs. C. Control or METTL17 edited cells expressing GFP, METTL17-FLAG, CYS^Mut^-FLAG or LYR^Mut^-FLAG constructs were grown for 24h in galactose, following which their viability was assessed (n=4–6). D. Immunoblots examining OXPHOS subunits or the loading control TUBULIN in Control or METTL17 edited cells expressing GFP, METTL17-FLAG, CYS^Mut^-FLAG or LYR^Mut^-FLAG constructs. E. Mitochondrial translation, as assessed by autoradiography after ^35^S-methionine/cysteine labeling, in Control or METTL17 edited cells expressing GFP, METTL17-FLAG, CYS^Mut^-FLAG or LYR^Mut^-FLAG constructs. All cells were treated with 200μg/mL emetine, and control cells in the last lane were also treated with 50μg/μL chloramphenicol. F. qPCR analysis of 12S levels in Control or METTL17 edited cells expressing GFP, METTL17-FLAG, CYS^Mut^-FLAG or LYR^Mut^-FLAG constructs (n=3 for all samples). G. Formaldehyde-linked RNA immunoprecipitation of the 12S to GFP, METTL17-FLAG, CYS^Mut^-FLAG or LYR^Mut^-FLAG proteins. Results were normalized to input construct and 12S levels (n=2 for all samples). All bar plots (except panel G) show mean ± SD. ****=p < 0.0001. Two-way ANOVA with Bonferroni’s post-test. See also Fig S4.

The striking loss of METTL17 protein levels in the absence of FXN resembles that of *bona-fide* Fe-S cluster containing proteins (Fig 1B) and made us wonder if perhaps METTL17 might also harbor an Fe-S cluster. Multiple sequence alignment (Fig 4A) of six METTL17 orthologs spanning bacteria, fungi, and humans revealed two striking features: (1) four conserved cysteine residues and (2) a tyrosine residue that forms part of an ‘LYR’ (leucine-tyrosine-arginine) motif in human METTL17. The former is likely a metal binding site, and indeed a recent cryoelectron microscopy (cryo-EM) structure of the trypanosomal METTL17 homologue revealed coordination of Zn^2+^ through these residues^59^. It is known that Zn^2+^ can replace a labile Fe-S cluster in aerobically purified proteins^60–62^. Of note, the METTL17 homologues do not contain the highly conserved CXXXCXXC motif typical of radical SAM proteins^63^. The LYR motif is often found in Fe-S cluster binding proteins^64^ and is proposed to engage the Fe-S cluster handoff machinery. The AlphaFold2.0 predicted structure of human METTL17^65,66^ shows that the conserved cysteines are spatially positioned so as to potentially form an Fe-S binding pocket while the LYR motif is surface accessible, strengthening the likelihood that these motifs are functional. Moreover, METTL17 remains stable following disruption of the cytosolic Fe-S biosynthesis machinery (Fig S4A), further hinting at a specific connection between loss of METTL17 and mitochondrial Fe-S cluster assembly. Collectively, these lines of evidence led us to hypothesize that METTL17 is a mitochondrial Fe-S cluster binding protein, and that perhaps this was previously missed given the labile nature of Fe-S clusters.

We asked whether these candidate Fe-S binding motifs were indeed important for the activity of METTL17. To this end, we generated three constructs for METTL17: (1) WT FLAG-tagged METTL17, (2) CYS^Mut^-FLAG mutant in which the conserved cysteines were changed to serines, (3) LYR^Mut^-FLAG mutant in which the LYR residues were changed to alanines. All three of these variant proteins were enriched in mitochondrial fractions (Fig 4B), demonstrating their proper subcellular targeting, although the two mutants were less abundant than the WT (as is typical of Fe-S cluster binding proteins lacking their cofactor). We next tested if these mutants could support mitochondrial translation in the absence of endogenous METTL17. METTL17-FLAG expression could rescue for loss of the endogenous gene as shown by reduced death in galactose and restored OXPHOS subunit levels, mitochondrial de-novo translation and 12S levels (Fig 4C–F and Fig 4SB–C). However, the CYS^Mut^-FLAG and LYR^Mut^-FLAG mutants failed to rescue any of the defects assayed. This would indicate that both motifs are crucial for METTL17’s role in supporting SSU activity. Of note, METTL17-FLAG expression served to boost mitochondrial translation and the steady state levels of multiple OXPHOS subunits (most notably in complexes I, II and IV) even above the GFP control, suggesting that it is a rate limiting factor in mitochondrial protein synthesis.

We next wondered whether the cysteine binding pocket and LYR motif were also required for METTL17 engagement with the SSU. We performed a formaldehyde RNA immunoprecipitation assay^67^ to test for the interaction between the various constructs and the 12S (Fig 4 G). In this assay, cells are lightly crosslinked, lysed, the protein of interest is immunoprecipitated and bound RNA is quantified by qPCR. We could observe that while METTL17-FLAG showed robust binding to the 12*S* as compared to a GFP control, the interaction between CYS^Mut^-FLAG or LYR^Mut^-FLAG and the 12*S* was diminished (even taking into account the lower basal expression levels for the constructs compared with 12*S*). By contrast, there was only marginal enrichment for 16*S* binding for these constructs when compared to the GFP control, and no notable difference between the WT and mutant forms of METTL17 (Fig S4D). These findings suggest that apart from structural stability, the putative Fe-S cluster in METTL17 is also important for contributing (directly or indirectly) to SSU and 12*S* binding.

### Biophysical studies confirm METTL17 harbors an Fe-S cluster

**Fig 5:**
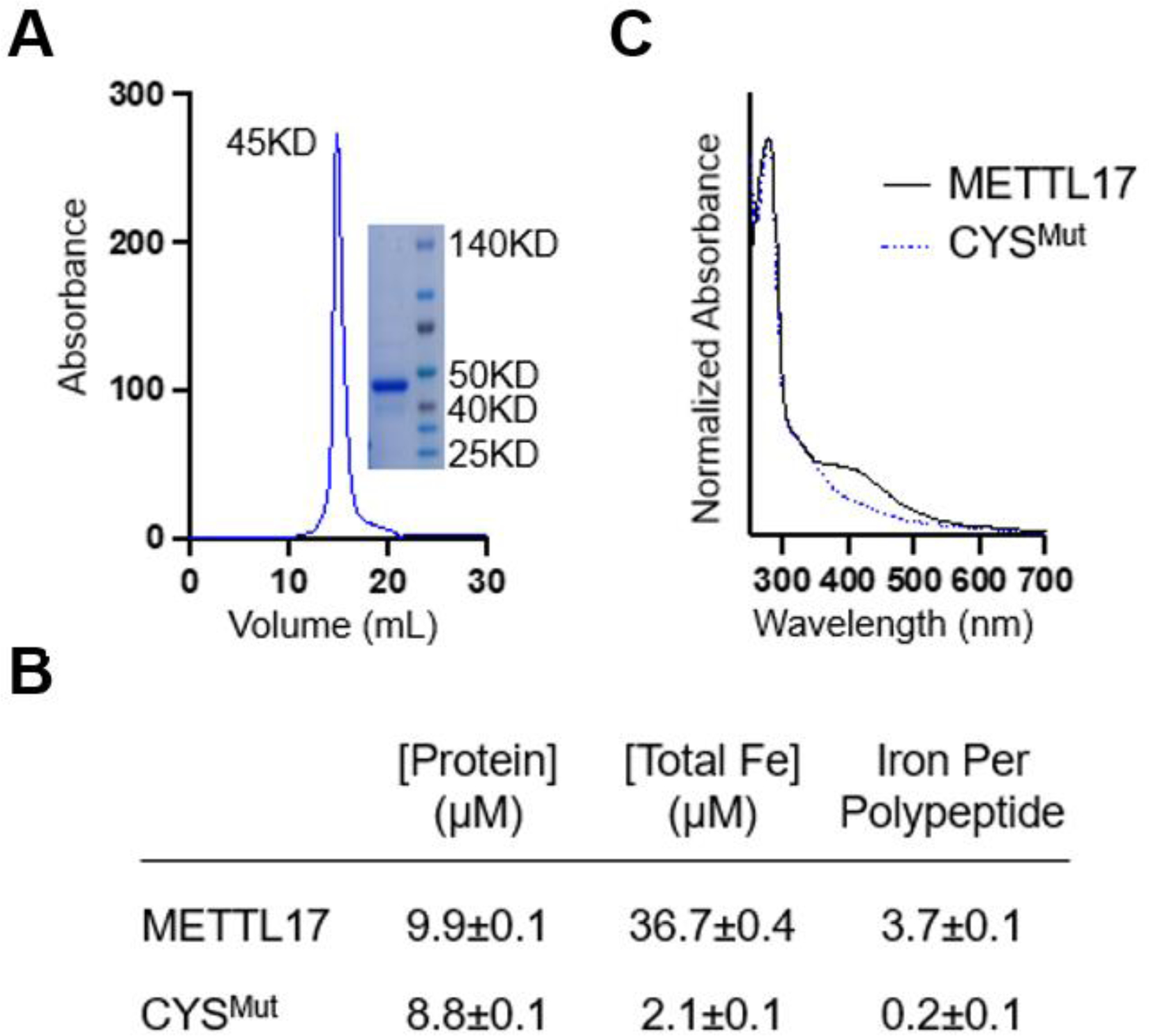
Human METTL17 expressed and purified from E. coli contains an Fe-S cluster. A. Gel filtration chromatography and SDS-PAGE analysis demonstrate that the purified METTL17 construct runs as a monomer near its predicted molecular weight of 50 kD. B. Iron content of purified METTL17and CYS^Mut^ as determined by bicinchoninic acid assay and inductively coupled plasma mass spectrometry. The CYS^Mut^ is a METTL17 variant in which the four cysteines predicted to coordinate the cluster are mutated to serine (C333S, C339S, C347S, and C404S). C. The UV-Vis absorption spectra of METTL17 exhibits a broad band around 420 nm, consistent with the presence of an [Fe_4_S_4_]^2+^ or an [Fe_3_S_4_]^+^ cluster; the latter is ruled out by EPR spectroscopy as described in the text. This band is lost in the spectrum of CYS^Mut^. For clarity, spectra were normalized to the intensity at 280 nm.

We then set out to study and analyze the purified human METTL17 under conditions that would be optimal to preserve any Fe-S cluster present. Affinity chromatography of METTL17-8xHIS gave a clean preparation of the protein that resulted in both a single band on an SDS-PAGE gel and a monodisperse analytical gel filtration peak corresponding to a molecular weight near 50 kDa (Fig 5A). Purified METTL17 precipitated upon exposure to air as is common for air-sensitive Fe-S proteins^62^. Inductively coupled plasma mass spectrometry (ICP-MS) analysis revealed the presence of 3.7 Fe ions per polypeptide (Fig 5B). In a variant in which the four cysteines predicted to coordinate the cluster (C333, C339, C347, and C404) were mutated to serines (‘CYS^Mut^’), almost all the iron was lost (0.2 Fe ions per polypeptide). The UV-Vis spectrum of METTL17 contains a broad charge-transfer band around 400 nm, suggesting the presence of either an [Fe _4_S_4_]^2+^ cluster or an [Fe_3_S_4_]^+^ cluster (Fig. 5C)^68–71^; this band is absent in the spectrum of the CYS^Mut^ sample. No electron paramagnetic resonance (EPR) signal was observed for the METTL17 sample between 5 and 50 K (data not shown), and addition of the reducing agent dithionite or the oxidizing agent indigodisulfonic acid also did not produce an EPR signal. The lack of an EPR signal in the as-isolated and oxidized samples rules out the presence of an [Fe_3_S_4_]^+^ cluster, which has a characteristic EPR signal^72^. Taken together, the Fe quantitation, UV-Vis spectroscopy, and EPR spectroscopy results are most consistent with the presence of an [Fe_4_S_4_]^2+^ cluster coordinated by C333, C339, C347, and C404 on human METTL17.

### The [Fe_4_S_4_]^2+^ cluster on METTL17 stabilizes its binding to the mitoribosomal small subunit prior to translation initiation

Next, we sought to directly visualize the Fe-S cluster in METTL17 using cryo-EM, with a focus on capturing it on the mitoribosome. We made initial attempts to study the human SSU-METTL17 complex, but METTL17 escaped detection as its binding is likely not stable enough. As the putative Fe-S cluster binding motif is conserved (Fig 4A), we used mitoribosomes from the yeast *S. cerevisiae*, which provide a suitable model for structural studies due to stabilizing rRNA expansion segments and protein extensions^73^ that would be amenable for studying assembly intermediates. Although previous fungi mitoribosome structures have been reported^74,75^, they did not investigate SSU assembly.

Reconstructions from the first cryo-EM dataset followed by 3D classification and refinement revealed two distinct classes, the empty and METTL17-bound states of the SSU (Fig S5). In the second dataset, we added a recombinant initiation factor mtIF3, and obtained three states: free SSU, METTL17-bound, mtIF3-bound. We then grouped particles from the two datasets and resolved the structures of the SSU, SSU-METTL17, and SSU-mtIF3 at an overall resolution of 2.3 Å, 2.6 Å, and 2.8 Å, respectively (Table S1). Compared to the previous report of *S. cerevisiae* SSU at 3.5 Å resolution^74^, the improved reconstruction allowed us to build a more accurate model. Specifically, we modelled extensions of the mitoribosomal proteins bS1m, uS2m, uS3m and uS7m as well as corrected the assignment of a guanosine diphosphate (GDP) to adenosine triphosphate (ATP) in the nucleotide pocket of the protein mS29 (S6 A-D).

**Fig 6:**
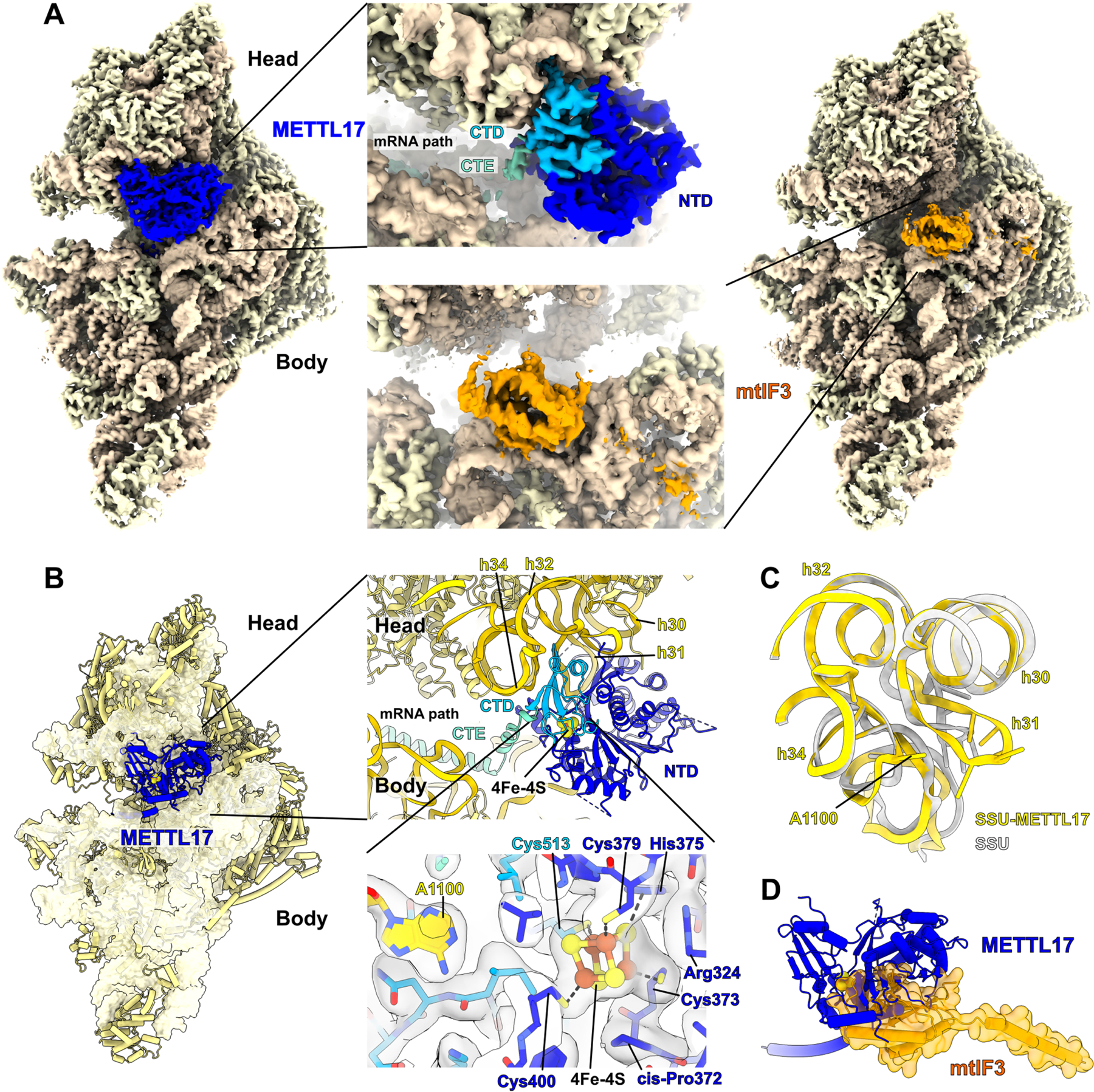
Cryo-EM structure of the yeast SSU-METTL17 complex and key functional elements. A. Cryo-EM density maps obtained for the SSU with METTL17 (left) or mtIF3 (right). B. Overall view of METTL17 on the SSU, and close-up views. Top close up shows the position of METTL17 (C-terminal domain, CTD sky blue; N-terminal domain, NTD blue) between the rRNA (yellow) of the head and body, while the C-terminal extension (CTE) occupies the mRNA path. Bottom close up shows the coordination of 4Fe-4S cluster by four cysteines, including Cys513 from the CTD, and related structural elements with their cryo-EM densities: flipped base A1100, a cis-proline, arginine that is within salt bridge distance, and a conserved histidine that can be involved in a transfer and ligation to the Fe-S unit. C. Conformational changes within the rRNA region h30–34 that is involved in METTL17 binding. Superimposed models of the SSU-METTL17 (yellow) with unbound state (grey). Sticks represent the rRNA residues responsible for the METTL17 interaction. D. Superposition of SSU-METTL17 with SSU-mtIF3 showing clashes of METTL17 (blue) with mtIF3 (orange surface representation). See also Table S1, Fig S5 and S6.

In the SSU-METTL17 complex, the 72-kDa factor is found in the cleft between the head and the body and its presence prevents a monosome formation (Fig 6A). In contrast to previously characterised SSU assembly factors^56^, METTL17 binds exclusively to the rRNA of the head (Fig 6B), and the binding involves both the N-terminal domain (NTD) and the C-terminal domain (CTD). The NTD is a Rossmann-fold methyltransferase domain, characterized by a seven-stranded beta-sheet core sandwiched by six alpha-helices, while the CTD is a unique fold that consists of a four-stranded beta-sheet with an alpha-helix. The comparison with the non-bound state shows that the association of METTL17 withdraws the rRNA helix h31 causing rearrangement of h32 and h34, and the entire head is rotated by approximately 3 Å up to open the tRNA binding cleft. Thus, h31 is more exposed, and the rRNA nucleotide A1100 in h34 is flipped out (Fig. 6C). In addition, the mitochondrial C-terminal extension fills the mRNA channel, blocking its premature binding.

The SAM motif Gly-X-Gly-X-Gly of *S. cerevisiae* METTL17 is disrupted with an Ala at the last Gly position and a Tyr at the first X position, suggesting that the methylation is not a conserved function of this protein in the process of the mitoribosome assembly. In fact, the 15S SSU rRNA in yeast is not methylated^76^ and no electron density was observed for a SAM cofactor. We identified an ordered density, adjacent to sulfhydryl groups of four cysteine residues Cys373, Cys379, Cys400, Cys513 (yeast numbering, equivalent to human Cys333, Cys339, Cys347, Cys404) (Fig 6B). The density corresponds to eight atoms organized in a cube, which is consistent with [Fe_4_S_4_]^2+^ cluster, where the four iron atoms are each bound by a terminal cysteine thiolate and three bridging sulfides. The unique feature of the embedded [Fe_4_S_4_]^2+^ cluster in our structure is that it brings together residues that are 140 amino acids apart, thus stabilizing the N- and C-terminal domains of METTL17 when it’s bound to the SSU. The resulting orientation of Cys400 and Cys513 forms a pocket, where rRNA base A1100 adapts, which contributes to a prominent binding of all the components together on the mitoribosome. Moreover, we identified a cis-proline Pro372 of METTL17 that orients the Cys373 side chain towards the [Fe_4_S_4_]^2+^ cluster and further supports the binding. Furthermore, we identified His375 and Arg324 within cluster interaction distance, suggesting a potential involvement in transfer and ligation of the [Fe_4_S_4_]^2+^ cluster. The AlphaFold2.0 predicted structure of the human METTL17^65,66^ is overall consistent with our model (Fig S6 E), and the superposition with the high-resolution structure of the human SSU^56^ suggests conserved interacting interfaces (Fig S6 F).

To further clarify the putative role of METTL17, we also formed a preinitiation complex by incubating the METTL17-containing SSU with a recombinant mtIF3 (the second dataset). A cryo-EM density map was then calculated, and we identified a new class containing a subset of 53,922 particles that was processed with a signal subtraction, followed by 3D classification using the mask for the mtIF3 - binding site (Fig S5). The map features the complex SSU-mtIF3, where the initiation factor occupies a position on the SSU that is similar to the human counterpart (Fig 6A and S6 G)^77^ and mutually exclusive with METTL17, as a superposition of the two states shows clashes between the factors (Fig 6D). Thus, the binding of the regulatory factor METTL17 is stabilized by the [Fe_4_S_4_]^2+^ cluster and precedes the initiation of translation.

### Forced expression of METTL17 rescues bioenergetic, but not growth defects, of FXN depleted cells

**Fig 7:**
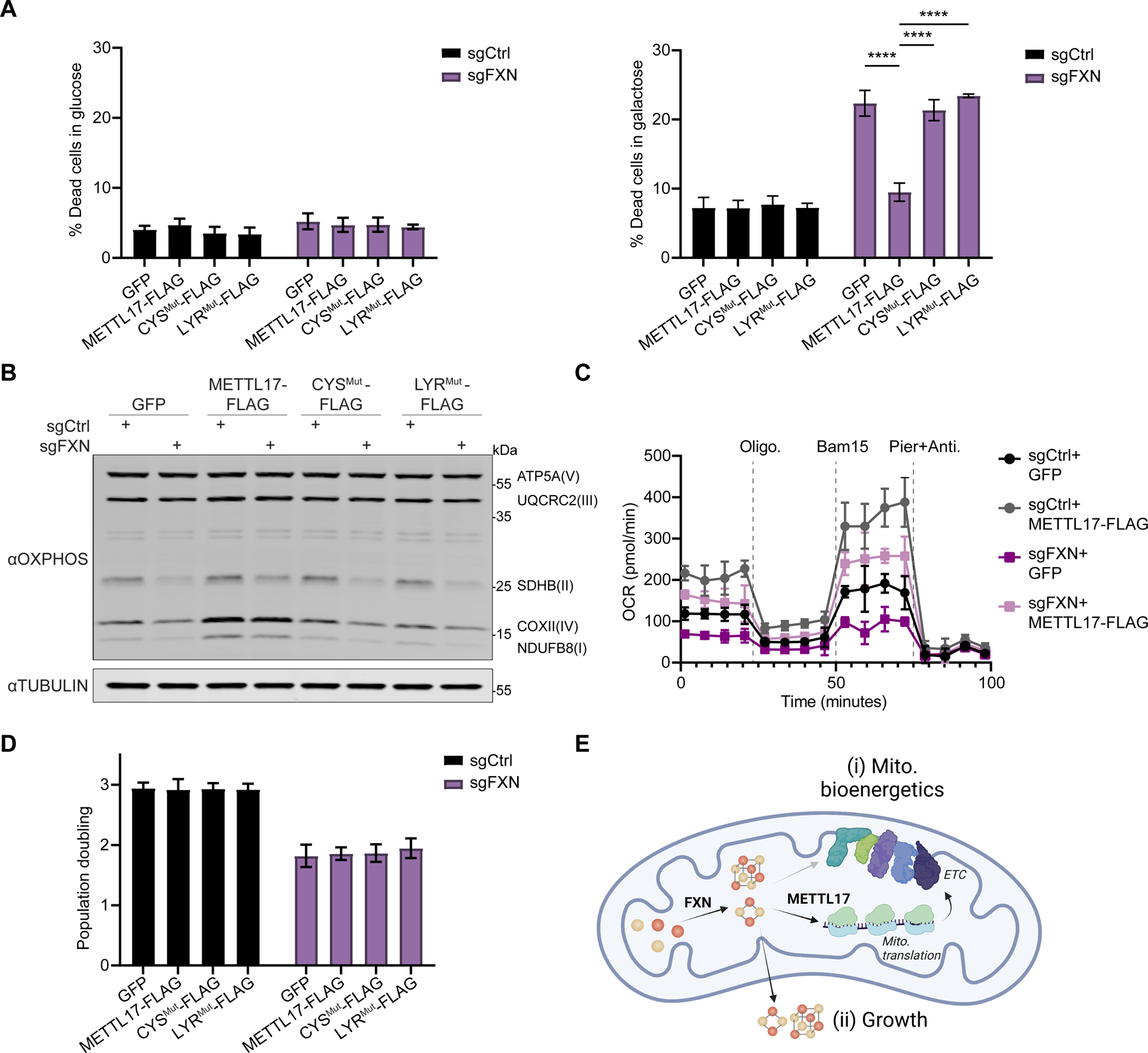
Overexpression of METTL17 restores the mitochondrial bioenergetics, but not growth, of FXN depleted human cells. A. Control or FXN edited cells expressing GFP, METTL17-FLAG, CYS^Mut^-FLAG or LYR^Mut^-FLAG constructs were grown for 24h in glucose (left) or galactose (right), following which their viability was assessed (n=4–6). B. Immunoblots examining OXPHOS subunits or the loading control TUBULIN in Control or FXN edited cells expressing GFP, METTL17-FLAG, CYS^Mut^-FLAG or LYR^Mut^-FLAG constructs. C. Oxygen consumption rate (OCR) of Control or FXN edited cells expressing GFP or METTL17-FLAG. Cells were sequentially treated with oligomycin, Bam15 and piericidin+antimycin. D. Population doubling over 72h of Control or FXN edited cells expressing GFP, METTL17-FLAG, CYS^Mut^-FLAG or LYR^Mut^-FLAG constructs (n=4 for all samples). E. FXN activates Fe-S cluster formation, which can be utilized to support (i) mitochondrial bioenergetics via formation of the electron transport chain (ETC) or (ii) cell growth and division. METTL17 is a key Fe-S cluster bearing modulator of mitochondrial bioenergetics, and its absence in FXN depleted cells accounts for much of the mito. bioenergetic defects observed in these cells. All bar plots show mean ± SD. **=p < 0.01, ****=p < 0.0001. Two-way ANOVA with Bonferroni’s post-test. See also Fig S7.

We sought to determine whether any of the phenotypic consequences of FXN deficiency could be prevented by forced expression of METTL17. We tested the effects of overexpressing METTL17, or its mutants, in *FXN* edited cells. What is striking is that we observed that the galactose-induced death of *FXN* edited cells could be reversed by the forced overexpression of METTL17-FLAG, but not CYS^Mut^-FLAG or LYR^Mut^-FLAG (Fig 7A). In contrast, overexpression of METTL17-FLAG in *ISCU* edited cells, depleted for the scaffold that is required for Fe-S cluster biosynthesis, only show a small, but significant, reduction in galactose-induced death (Fig S7A–B). In line with these findings, we showed that the loss of various OXPHOS protein subunits and the lowered respiratory rates of FXN edited cells were restored to WT levels by overexpression of METTL17-FLAG (Fig 7B–C and Fig S7C–D), but not in ISCU edited cells (Fig S7E). Thus, it seems that the bioenergetic defect of *FXN* depleted cells could largely be reverted by re-expressing one mitochondrial Fe-S cluster containing protein: METTL17. However, residual Fe-S cluster biosynthesis is required for this restorative effect, as it does not occur in the absence of ISCU or when a mutant METTL17 (that cannot bind an Fe-S cluster) is used.

Next, we wondered if other cellular defects linked to attenuated Fe-S cluster biosynthesis could be rescued by METTL17 overexpression, as this cofactor is required for other processes both within the mitochondria (e.g. lipoic acid synthesis) and outside of it (e.g, nuclear DNA maintenance, repair and nucleotide synthesis). When we tested the growth of *FXN* edited cells upon METTL17 overexpression, however, we saw no benefit (Fig 7D). Moreover, the nuclear Fe-S binding protein POLD1 remained depleted in *FXN* edited cells irrespective of METTL17 overexpression (Fig S7F). Finally, the mitochondrial Fe-S cluster containing protein, LIAS, which is translated by the cytosolic ribosome, was reduced regardless of METTL17 overexpression (Fig S7G). Thus, METTL17 overexpression is specifically restoring mitochondrial respiration under cluster-limited (but not cluster-void) conditions, likely via its role in regulating mitochondrial translation.

Together, these data demonstrate that the growth and bioenergetic defects observed in *FXN* edited cells can be uncoupled from each other through the expression of an endogenous factor— the Fe-S cluster containing protein METTL17. Fe-S clusters that are made in the mitochondria are directed to distinct routes: one for local use within mitochondria supporting bioenergetic demand vs. one for export to the cytosol to support growth (Fig 7E). METTL17 is a rate limiting factor in sustaining mitochondrial translation, and ergo respiratory complex formation, whose presence is tightly linked to Fe-S cluster levels.

## Discussion

Here we have made the discovery that METTL17, a previously described putative SSU maturation factor, harbors a hitherto unrecognized [Fe_4_S_4_] cluster that is critical for its function in protein synthesis. We show that METTL17 deficiency lies downstream of *FXN* loss and appears to be a key effector of impaired mitochondrial translation. Re-expressing METTL17 is sufficient to rescue the bioenergetic, but not growth, phenotypes associated with *FXN* deficiency. These findings demonstrate that in Fe-S cluster limiting conditions, formation of the respiratory chain is primarily restricted by cluster-regulated mtDNA translation, via METTL17.

Our proteome-wide analysis indicates that nearly all the Fe-S cluster containing proteins we could detect are de-stabilized when the Fe-S cluster supply is reduced. While it has long been known that one function of Fe-S clusters (in addition to their many roles in electron transfer, oxyanion binding, etc.) is protein stability^24^, our findings show that in fact this is a nearly universal role of the cluster. Previous proteomic profiling of FA patient cells has described deficits in some, but not all, Fe-S cluster containing proteins^78^. However, it is likely that a near total depletion of *FXN*, such as is generated by CRISPR editing, is not observed in patients.

Our structural studies have revealed that the [Fe_4_S_4_]^2+^ cluster stabilizes METTL17 and assists in its coupling to the rRNA of the mitoribosomal small subunit during the assembly process. Comparative sequence analysis indicates that the [Fe_4_S_4_]^2+^ cluster binding residues on METTL17 are more conserved than the SAM binding motif. When examining the METTL17 ortholog *Dictyostelium discoideum*, a member of the Amoebozoa outgroup of Metazoa and Fungi, we find that both the SAM motif and the four cysteine residues coordinating Fe-S cluster binding are present indicating that this is likely the more ancient form of the protein. It is notable that radical SAM enzymes are methyltransferases that harbor Fe-S clusters required for catalysis. However, the sequence alignment and cryo EM structure of *S. cerevisiae* METTL17 argue against METTL17 being a radical SAM enzyme, given the lack of the CXXXCXXC motif ^63^ and the long distance between its Fe-S cluster and the SAM binding motif. The obtained structure of the SSU-METTL17 complex allows us to put METTL17 in the context of the dynamic SSU assembly, extending its part in the view of the mitoribosome formation. Comparison with structural studies of earlier assembly intermediate states ^56^ suggests that METTL17 can be accommodated on the SSU in the presence of RBFA, and we also show that METTL17 is replaced by the initiation factor mtIF3. Based on these analyses, we conclude that METTL17 facilitates a productive mitorobosome-assembly pathway at relatively late stages, which reflects the structural importance of the [Fe_4_S_4_]^2+^ bound METTL17 for the biological function of the mitoribosome biogenesis.

Our data indicates that METTL17 operates as an Fe-S cluster “checkpoint” for protein synthesis. The mitochondrial respiratory chain is rich with Fe-S clusters, embedded within complexes I, II and III ^23,24^. Our work shows that mitochondrial translation is dependent on METTL17 being present with an intact Fe-S cluster for proper ribosome assembly. We have shown that METTL17 is highly labile in the absence of Fe-S clusters, and moreover, that METTL17 is limiting for mitochondrial translation. Hence, METTL17 can ensure that mitochondrial translation proceeds only when Fe-S cluster levels are replete. As solvent exposed Fe-S clusters are extremely labile to reactive nitrogen and oxygen species^60,79^, METTL17 could serve as a “kill switch” for turning off the production of mitochondrial machinery in unfavorable environments, enabling sub-organelle regulation of mitochondrial protein synthesis to match local redox conditions^80^. Indeed, recent work in purified mitochondria has demonstrated that METTL17 is highly sensitive to oxidative stress^81^, indicating that METTL17 levels might be primarily controlled by its half -life and could in theory act as a local translational regulatory element.

Our findings indicate that at least in cultured cells, *FXN* deficiency results in a previously under-appreciated deficit in mitochondrial protein synthesis, which appears to be largely downstream of METTL17. Although OXPHOS deficiency, specifically loss of complexes I and II, have long been appreciated to be features of FA samples^29,30^, to the best of our knowledge, no prior report has documented defects in mitochondrial protein synthesis. Indeed, complex I seems to be doubly vulnerable during conditions of limited Fe-S cluster availability, as it is depleted both due to attenuated mitochondrial translation and co-factor availability. Future efforts should be aimed at determining whether patient derived specimens show any evidence of impaired mitochondrial translation. Recent work has identified Fe-S cluster proteins involved in mitochondrial translation embedded in tRNA maturation^57,82^ as well as in the mitoribosome^83,84^. However, overexpression of METTL17 seems to be sufficient to restore the respiratory defects of FXN depleted cells, suggesting it may be the primary limiting factor for mitochondrial protein synthesis in this context.

A curious feature of METTL17 is that its expression uncouples the bioenergetic versus proliferative effects of Fe-S clusters, activating the former without affecting the latter. In animal cells, *de novo* Fe-S cluster biosynthesis begins inside of mitochondria, and this supply is then conveyed within the organelle and also exported to the cytosol and nucleus, where it supports various anabolic processes^7,10,24^. Both evolutionary and genetic lines of evidence have shown that the essentiality of Fe-S cluster biosynthesis can be attributed to extra-mitochondrial demand for these cofactors^85–89^. It is interesting to speculate whether additional, yet unidentified, factors act as “checkpoints” when routing Fe-S clusters to extra-mitochondrial proliferative pathways. The ability to uncouple these routes by expressing METTL17 could be useful for dissecting the pathogenesis of FA, since at present, we do not know which is dominant in the etiopathogenesis: bioenergetic defect or growth defect. If the bioenergetic defects predominate *in vivo*, boosting METTL17 could hold therapeutic potential. Moreover, this property of METTL17 suggests it could represent an interesting therapeutic target, not only for FA. In principle, other inherited OXPHOS disorders- independent of genetic etiology- may also benefit from boosting mitochondrial protein synthesis via METTL17 overexpression.

## Limitations of the Study

We performed our proteomic analysis on FXN depleted cells as a screen, and hence, it was only performed in duplicate. We acknowledge it has limited statistical power. This study has demonstrated that overexpression of METTL17 is sufficient to restore the bioenergetic defects of FXN edited human cells dividing in culture. One of the key challenges going forward will be to test whether this also holds true in human biopsy material or in animal models of FA. It will also be interesting to see if all tissue types will similarly benefit from METTL17 overexpression in states of frataxin deficiency. Due to technical limitations, we were unable to determine the degree of METTL17 overexpression that restores the respiratory capacity of FXN edited cells.

## STAR Methods

### Resource availability

#### Lead Contact

Further information and requests for resources and reagents should be directed to and will be fulfilled by the lead contact, Vamsi K. Mootha (vamsi@hms.harvard.edu).

#### Materials availability

Materials generated in this study are available from the lead contact upon reasonable request.

### Data and code availability

- The cryo-EM density maps and atomic coordinates have been deposited in the Electron Microscopy Data Bank (EMDB) and Protein Data Bank (PDB) under accession codes EMD-16966, EMD-16967, EMD-16968 and 8OM2, 8OM3, 8OM4 respectively. Sequencing data are available at GEO with identifier GSE242192. Proteomics data are available via ProteomeXchange with identifier PXD045443. All deposited datasets will be publicly available upon publication. The remaining data has been included in this manuscript as supplementary files.
- This paper does not report original code.
- Any additional information required to reanalyze the data reported in this paper is available from the lead contact upon request.

### Experimental model and study participant details

#### Cell Lines

K562 (female), HEK293T (female) and A549 (male) cells were obtained from the ATCC and maintained in DMEM (GIBCO) with 25 mM glucose, 10% fetal bovine serum (FBS, Invitrogen), 4mM Glutamine, 1 mM sodium pyruvate, 50 μg/mL uridine, and 100 U/mL penicillin/ streptomycin under 5% CO_2_ at 37°C. When necessary, K562 cells were selected with 2 μg/ml puromycin (GIBCO) or 500ug/ml Geneticin (GIBCO), HEK293T and A549 cells were selected with 1 μg/ml puromycin (GIBCO). Cell lines were authenticated by STR profiling (ATCC). Cells were tested to ensure absence of mycoplasma by PCR-based assay once every 3 months. For experiments involving 1% oxygen, cells were placed in 37°C incubators, attached to a nitrogen supply which pulsed N2 and maintained 1% O_2_ and 5% CO_2._ Polyclonal CRISPR-Cas9 edited cells were used throughout.

### Method details

#### Plasmids

Individual sgRNAs were cloned into pLentiCRISPRv2 (Addgene 52961)^91^. For genetic interaction assays, cells were infected with pRDA_186 plasmid (Addgene 133458), a gift from John Doench (Broad Institute), bearing guides against a control locus or FXN. C-terminally FLAG tagged, codon optimized cDNAs were cloned in pLYS6, bearing a Neomycin selection cassette, using the NheI and EcoRI sites. All plasmids were verified by sequencing. pMD2.G (Addgene 12259) and psPAX2 (Addgene 12260) were used for lentiviral packaging.

#### Lentivirus production

2.5 x10^6^ HEK293T cells were seeded in 5ml in a T25cm^2^ flask (one flask per lentivirus). The following day the cells were transfected with 1ml of transfection mixture per well. The transfection mixture contained 25 μl Lipofectamine 2000 (Thermo Fisher Scientific), 3.75μg psPAX2, 2.5μg pMD2.G, 5μg of the lentiviral vector of interest and Opti-MEM medium (GIBCO) up to 1ml. The mixture was incubated at room temperature for 20 min before adding it to cells. 6h following transfection, the media was replaced with fresh DMEM. Two days after transfection, media was collected, filtered through a 0.45um filter and stored at −80C.

#### Proteomics

sgCtrl and sgFXN K562 cells were grown in duplicate flasks for 10 days. Quantitative proteomics was performed at the Thermo Fisher Scientific Center for Multiplexed Proteomics (Harvard).

##### Sample Preparation for Mass Spectrometry

Samples were prepared essentially as previously described^92,93^. Following lysis, protein precipitation, reduction/alkylation and digestion, peptides were quantified by micro-BCA assay and 100μg of peptide per sample were labeled with TMT reagents (Thermo-Fisher) for 2hrs at room temperature. Labeling reactions were quenched with 0.5% hydroxylamine and acidified with TFA. Acidified peptides were combined and desalted by Sep-Pak (Waters).

##### Basic pH reversed-phase separation (BPRP)

TMT labeled peptides were solubilized in 5% ACN/10 mM ammonium bicarbonate, pH 8.0 and separated by an Agilent 300 Extend C18 column (3.5μm particles, 4.6 mm ID and 250 mm in length). An Agilent 1260 binary pump coupled with a photodiode array (PDA) detector (Thermo Scientific) was used to separate the peptides. A 45 minute linear gradient from 10% to 40% acetonitrile in 10 mM ammonium bicarbonate pH 8.0 (flow rate of 0.6 mL/min) separated the peptide mixtures into a total of 96 fractions (36 seconds). A total of 96 Fractions were consolidated into 24 samples in a checkerboard fashion, acidified with 20 μL of 10% formic acid and vacuum dried to completion. Each sample was desalted via Stage Tips and re-dissolved in 5% FA/ 5% ACN for LC-MS3 analysis.

##### Liquid chromatography separation and tandem mass spectrometry (LC-MS3)

Proteome data were collected on an Orbitrap Eclipse mass spectrometer (ThermoFisher Scientific) coupled to a Proxeon EASY-nLC 1200 LC pump (ThermoFisher Scientific). Fractionated peptides were separated using a 180 min gradient at 500 nL/min on a 35 cm column (i.d. 100 μm, Accucore, 2.6 μm, 150 Å) packed in-house. MS1 data were collected in the Orbitrap (120,000 resolution; maximum injection time 50 ms; AGC 4 × 105). Top 10 precursors with charge states between 2 and 5 were required for MS2 analysis, and a 90 s dynamic exclusion window was used. MS2 scans were performed in the ion trap with CID fragmentation (isolation window 0.5 Da; Rapid; NCE 35%; maximum injection time 35 ms; AGC 1.5 × 104). An on-line real-time search algorithm (Orbiter) was used to trigger MS3 scans for quantification^94^. MS3 scans were collected in the Orbitrap using a resolution of 50,000, NCE of 55%, maximum injection time of 150 ms, and AGC of 1.5 × 105. The close out was set at two peptides per protein per fraction(Schweppe *et al.*, 2020).

##### Data analysis

Raw files were converted to mzXML, and monoisotopic peaks were re-assigned using Monocle^95^. Searches were performed using SEQUEST^96^ against a human database downloaded from Uniprot in 2014. We used a 50 ppm precursor ion tolerance and 0.9 Da product ion tolerance for MS2 scans collected in the ion. TMT on lysine residues and peptide N-termini (+229.1629 Da) and carbamidomethylation of cysteine residues (+57.0215 Da) were set as static modifications, while oxidation of methionine residues (+15.9949 Da) was set as a variable modification.

Each run was filtered separately to 1% False Discovery Rate (FDR) on the peptide-spectrum match (PSM) level. Then proteins were filtered to the target 1% FDR level across the entire combined data set. For reporter ion quantification, a 0.003 Da window around the theoretical m/z of each reporter ion was scanned, and the most intense m/z was used. Reporter ion intensities were adjusted to correct for isotopic impurities of the different TMT reagents according to manufacturer specifications. Proteins were filtered to include only those with a summed signal-to-noise (SN) ≥ 100 across all TMT channels. For each protein, the filtered peptide TMT SN values were summed to generate protein quantification values. To control for different total protein loading within a TMT experiment, the summed protein quantities of each channel were adjusted to be equal within the experiment. Data was analyzed as Log10 fold-change of each protein.

#### Mito translation assay

2.5x10^6^ cells expressing the corresponding sgRNAs were washed in PBS and incubated for 30 min in 1 mL of labeling medium (10% dFBS, 1 mM sodium pyruvate, and 50 μg/mL uridine in DMEM without methionine/cysteine; Life Technologies). Emetine (Sigma) was added to a final concentration of 200 μg/mL, and cells were incubated for 5 min before addition of 200μCi 35S-labeled methionine/cysteine mixture (PerkinElmer) and incubation for 1 hr at 37°C. Cells were recovered and washed twice in PBS before lysis in RIPA buffer (50 mM Tris-HCl pH 7.5, 150 mM NaCl, 1.0% NP-40, 0.5% sodium deoxycholate, 0.1% SDS, 1x protease and phosphatase inhibitor (Cell Signaling), and 250 units/ml benzonase nuclease (Sigma)). 40 μg of total proteins were loaded on a 10%–20% SDS-PAGE (Life Technologies) and transferred to a nitrocellulose membrane, 0.45 μm (BioRad). Total protein in each lane was assessed by ponceau S (ThermoFisher) staining and recorded before autoradiography. In all experiments, a replicate of the control lane was treated with chloramphenicol (50 μg/mL) to ensure the mitochondrial origin of the 35S signal. The name associated with each band is based on the predicted molecular weight of mtDNA encoded proteins. These assignments are also in line with prior studies^97^ demonstrating the absence of single nascent mitochondrial polypeptides of comparable molecular weight caused by particular point mutations or deletions in the mtDNA.

#### Genetic interaction screening

##### Screening

K562 cells were infected with pRDA_186 lentiviral vectors, which express sgRNA against a control locus or FXN, blasticidin resistance from the PGK promoter, and a 2A site-expressing EGFP. Cells were sorted for low GFP expression and expanded.

For the screen, the all-in-one Brunello barcoded library was utilized. This library contains 77,441 sgRNA; an average of 4 guides per gene and 1000 non–targeting control guides. Infections were performed in distinct duplicate at a predetermined MOI of ~0.5 in 12-well plates with 5μg/mL polybrene supplementation. Cells were infected for 2h under centrifugation at 1000 x g, incubated for 22h under standard culturing conditions and pooled 24 h post-centrifugation. Infections were performed with 6.5x10^7^ cells per replicate, in order to achieve a representation of at least 300 cells per sgRNA following puromycin selection. 48 hours after infection, cells were selected with puromycin for 2 days to remove uninfected cells. Cells were passaged in fresh media every 2–3 days. Cells were harvested 11 days after initiation of treatment.

For all screens, genomic DNA (gDNA) was isolated using the XL Maxi NucleoSpin Blood kit (Macherey-Nagel) according to the manufacturer’s protocol. PCR and sequencing were performed as previously described^98,99^. Briefly, gDNA was divided into 100μL reactions such that each well had at most 10 μg of gDNA. Per 96 well plate, a master mix consisted of 75 μL ExTaq DNA Polymerase (Clontech), 1000 μL of 10x Ex Taq buffer, 800 μL of dNTP provided with the enzyme, 50 μL of P5 stagger primer mix (stock at 100 μM concentration), and 2075 μL water. Each well consisted of 50 μL gDNA plus water, 40 μL PCR master mix, and 10 μL of a uniquely barcoded P7 primer (stock at 5 μM concentration). PCR products were purified from an agarose gel, using QIAquick Gel Extraction kit (QIAGEN). Samples were sequenced on a MiSeq (Illumina).

##### Analysis

Analysis of genetic interactors was performed as previously described^100^. Briefly, raw sgRNA read counts were normalized to reads per million and then log2 transformed using the following formula:

Log2(Reads from an individual sgRNA/Total reads in the sample x 10^6^ + 1)

Log2 fold-change of each sgRNA was determined relative to guide abundance in the lentiviral library. The replicates were paired throughout the course of the screens.

For each gene and for each replicate, the mean log2 fold-change in the abundance of all 4 sgRNAs was calculated. Log2 fold-changes were averaged by taking the mean across replicates. For each treatment, a null distribution was defined by the 3,726 genes with lowest expression (log2 FPKM = −7) according to publicly available K562 RNA-seq dataset (sample GSM854403 in GEO series GSE34740). To score each gene, its mean log2 fold-change across replicates was Z-score transformed, using the statistics of the null distribution defined as above.

#### Polyacrylamide gel electrophoresis and protein immunoblotting

2–5 x10^6^ cells were harvested, washed in cold PBS and lysed for 10min on ice in RIPA lysis buffer (50 mM Tris-HCl pH 7.5, 150 mM NaCl, 1.0% NP-40, 0.5% sodium deoxycholate, 0.1% SDS, 1x HALT protease phosphatase inhibitor (Thermo Fisher Scientific), and Pierce Universal Nuclease for Cell Lysis (Thermo Fisher Scientific). Lysates were further clarified by centrifugation for 10min at 16000 x g at 4C. Protein concentration was measured using Pierce 660nm Protein Assay (Thermo Fisher Scientific). 30ug was loaded per well. Electrophoresis was carried out on Novex Tris-Glycine 4–20% or 10–20% gels (Life Technologies) before transfer on a nitrocellulose membrane, 0.45 μm (BioRad). Membranes were blocked for 60mins with Odyssey Blocking Buffer (LI-COR Biosciences) at RT. Membranes were then incubated with primary antibody, diluted in 3%BSA, for 1h at RT or overnight at 4C. Membranes were then washed at RT 5 times in TBST for 5 mins. The membrane was incubated with goat α-rabbit or α-mouse conjugated to IRDye800 or to IRDye680 (LI-COR Biosciences), diluted in 5% milk, for 1h at RT. Membranes were washed 3 times in TBST for 5mins and were scanned for infrared signal using the Odyssey Imaging System (LI-COR Biosciences). Band intensities were analyzed with Image Studio Lite (LI-COR Biosciences).

#### Cell viability assay in galactose vs. glucose

To measure viability in galactose vs glucose, cells were washed in PBS, counted and an equal number of cells was seeded in culture media containing 25mM glucose or 25mM galactose with 10% dialyzed FBS. 24h later, cells were collected and viable cells were determined using a Vi-Cell Counter (Beckman).

#### qPCR

2.5x10^6^ cells were collected per sample. RNA was extracted from total cells with an RNeasy plus kit (QIAGEN) and DNase-I digested before murine leukemia virus (MLV) reverse transcription using random primers (Promega). qPCR was performed using the TaqMan technology (Life Technologies), using probes Hs02596859_g1 (12S), Hs02596860_s1 (16S), Hs00224159_m1 (METTL17) and Hs00427620_m1 (TBP). All data were normalized to TBP.

#### Formaldehyde RNA immunoprecipitation (fRIP) assay to METTL17-12S binding

fRIP assay was performed as previously described^67^ with some minor changes. Briefly, 5 x10^6^ K562 cells were cross-linked with 0.1% formaldehyde (Polysciences, 04018) and slowly rotated for 10 min at room temperature. To quench this reaction, glycine was added to a final concentration of 125mM and incubated for 5 min at room temperature with rotation. Cells were collected and spun down at 300g for 5min at 4°C. The supernatant was removed and the pellet was washed with 1mL cold PBS. The supernatant was removed and the pellet was resuspended in 1ml RIPA buffer (buffer (50 mM Tris-HCl pH 7.5, 150 mM NaCl, 1.0% NP-40, 0.5% sodium deoxycholate, 0.1% SDS, 1x protease and phosphatase inhibitor (Cell Signaling), and 250 units/ml benzonase nuclease (Sigma)). The cells were sonicated for 3 times each (10 sec per cycle, amplitude 7 μm) with a sonifier (Branson). Samples were then further lysed on ice for 10 min. To remove cell debris, the extract was centrifuged at 12,000g for 10 min at 4°C. 150 μL of the supernatant was removed and stored at −20°C (input sample), and the remainder of the sample was used for precipitation. Anti-FLAG^®^ M2 magnetic beads (Sigma) were equilibrated 3 times with 1 mL RIPA buffer. 20μL of anti-FLAG beads were used for precipitation. The IP was performed overnight at 4°C on a spinning wheel.

Beads were recovered after extensive washing in RIPA buffer. After the last washing step, the supernatant was removed, 100μL of RNAse free water with RNAsin (40 U/mL) was added and samples incubated in a thermomixer (Eppendorf) at 300 rpm for at 55°C for 1h for decrosslinking. 1ml of Trizol (Invitrogen) was then added to each sample, and RNA extraction was performed per the manufacturer’s recommendations. RNA samples were subsequently quantified for 12S levels by qPCR, and the values were normalized to input 12S and FLAG tagged protein levels.

#### mtDNA copy number

1x10^5^ cells from each condition (n = 3) were harvested and lysed in 100μL mtDNA lysis buffer (25mM NaOH, 0.2mM EDTA) before incubation at 95°C for 15min. 100μL of 40mM Tris-HCl pH 7.5 was added to neutralize the reaction on ice. Samples were diluted 50x and the ratio between mitochondrial and nuclear DNA was determined using a custom Taqman based assay and qPCR using a CFX Opus 384 quantitative PCR machine (Biorad). Relative mtDNA abundance was determine using the ΔΔCt method.

#### Mitostring

2x10^6^ cells were collected per sample. RNA was extracted from total cells with an RNeasy kit (QIAGEN). MitoString is a mitochondrial-specific version of NanoString and was performed as previously described (Wolf and Mootha, 2014). All counts were normalized to TUBB.

#### Mitochondrial isolation

5x10^7^ cells were harvested, washed in PBS, and washed once with 10 mL of MB buffer (210 mM mannitol, 70 mM sucrose, 10 mM HEPES-KOH at pH 7.4, 1mM EDTA, protease/phosphatase inhibitor). Cells were resuspended in 1 mL of MB buffer supplemented with 1x HALT protease phosphatase inhibitor (Thermo Fisher Scientific) and transferred to 2 mL glass homogenizer (Kontes). Cells were broken with ~35 strokes of a large pestle on ice. Sample volume was then increased to 6 mL with MB buffer. The mixture was centrifuged at 2,000xg for 5 min and the pellet (nuclei and intact cells) was discarded. The supernatant was centrifuged at 13,000xg for 10 min at 4°C. The mitochondrial pellets were washed with MB buffer once, and resuspended in RIPA lysis buffer.

#### Oxygen Consumption

1.50x10^5^ K562 cells per well were plated on a Seahorse plate coated with Cell-Tak Cell and Tissue Adhesive (Corning Life Sciences) in XF DMEM (Agilent) containing 5.55mM glucose, 4mM Glutamine, 1mM sodium pyruvate, and oxygen consumption was recorded using a Seahorse XF96 Analyzer (Seahorse Biosciences). Each measurement was performed over 6 min after a 3 min mix and a 3 min wait period. Basal measurements were collected 4 times, followed by 4 measurements after addition of oligomycin (final concentration 2μM), followed by 4 measurements after addition of Bam15 (final concentration 3μM), followed by 4 measurements after addition of Piericidin A+ Antimycin A (final concentration 0.5 μM).

#### Proliferation assays

Cell proliferation assays were performed 7–8 days following lentiviral infection. Cells were seeded at an initial density of 1×10^5^ cells/mL and cultured for 3 days. Viable cell number was then determined using a Vi-Cell Counter (Beckman).

#### Expression and purification of METTL17-8XHIS

A human METTL17 construct was designed for expression in E. coli in which the N-terminal mitochondrial targeting sequence was removed and a C-terminal TEV site and 8X HIS-tag were introduced. The METTL17-8XHIS gene was codon optimized for *E. coli* and ligated into a pET-30a(+) expression vector. The protocol for overexpression and purification of METTL17-8XHIS was adapted from that used for the yeast homolog^101^. METTL17-8XHIS was overexpressed in OverExpress C41(DE3) cells in LB supplemented with 50 μM FeCl3 and 300 μM L-cysteine at 37ºC. Cells were grown until an OD600 of 0.8 and then expression was induced with 1mM IPTG. Cells were pelleted after 4 hours, then frozen and stored in liquid nitrogen until purification. At the time of purification, pellets were thawed and resuspended in Buffer A containing 500 mM NaCl, 5 mM imidazole, 5% glycerol, 1 mM TCEP, 40 mM HEPES pH 7.4, and supplemented with lysonase, benzonase, DNase, ribonuclease A, Roche Complete protease, and PMSF. The cells were lysed using sonication and clarified through centrifugation at 40,000 × g for one hour. METTL17-8XHIS was then purified over TALON cobalt resin. After washing the column with Buffer A containing 40 mM imidazole, the protein was washed with 30 CVs of a solution containing 2.5 M NaCl, 5% glycerol, 1 mM TCEP, 40 mM HEPES pH 7.4, and 0.1 mg/mL ribonuclease A and DNase I to remove nucleic acid contamination. The protein was eluted with Buffer A containing 300 mM imidazole and then run over a PD10 column into a buffer containing 500 mM NaCl, 5% glycerol, 1 mM TCEP, and 40 mM HEPES pH 7.4. Sizing of the protein was done over a Superdex 200 increase 10/300 GL column. The variant CYS^Mut^-8xHIS containing the mutations C333S, C339S, C347S, and C404S was expressed and purified using the same method.

#### UV/vis spectroscopy

UV/vis spectra were collected at room temperature with a Cary 50 UV/vis spectrophotometer using a 1 cm pathlength quartz cuvette. Aerobically purified METTL17-8XHIS or CYS^Mut^-8xHIS was transferred into a Coy Labs glovebox (<5 ppm O_2_). The protein solution was equilibrated to the glovebox atmosphere for at least 30 min on ice before it was centrifuged for 3 min at 14,000 × g and room temperature, then buffer-exchanged into an anaerobic buffer containing 25 mM HEPES, pH 7.5, 10% glycerol, 500 mM NaCl using a PD-10 column (GE Healthcare). Samples were diluted (if necessary) with the same buffer and transferred to a quartz cuvette.

#### EPR spectroscopy

Electron paramagnetic resonance (EPR) spectra were recorded on a Bruker EMX spectrometer at 9.37 GHz as frozen solutions. Samples were transferred to a quartz tube (made from clear fused quartz tubing of 3 mm I.D. & 4 mm O.D. from Wale Apparatus) in a Coy Labs glovebox (<5 ppm O_2_), capped with a rubber septum, and frozen in liquid N_2_ outside the glovebox. Each sample contained approximately 200 μL of 30 μM aerobically purified METTL17-8XHIS with/without 2 mM Na_2_S_2_O_4_ as a reductant or 0.5 mM indigodisulfonic acid as an oxidant.

#### Fe analysis

Protein concentrations were determined by BCA assay using bovine serum albumin (Thermo Scientific) as standards^102^. Fe concentrations were determined by inductively coupled plasma mass spectrometry (ICP-MS). ICP-MS data were recorded on an Agilent 7900 ICP-MS instrument. Samples were prepared by first digesting the protein in 70% nitric acid (TraceMetalTM Grade, Fischer) at 60 °C, and then diluting it with Milli-Q water such that the final concentration of nitric acid is 2%. Standards for Fe were prepared from a 100 ppm transition metal standard solution (Specpure, Thermo Fischer). All samples and standards contained 1 ppb Tb (final concentration) as an internal standard.

#### Mitoribosome purification for structural studies

Yeast mitochondria were harvested as previously described^73^. Four litres of *S. cerevisiae* were grown in YPG media (1% yeast extract, 2% peptone, 3% glycerol) until an optical density at 600 nm (OD_600_) of 1.8. The cells were then centrifuged at 4,500 × *g,* for 9 min, the pellet washed with pre-cooled distilled water and further centrifuged for 15 min at 4,500 × *g.* The pellet was resuspended in pre-warmed dithiothreitol (DTT) buffer (100 mM Tris-HCl pH 9.3, 10 mM DTT). The cells were pelleted by centrifugation at 3,500 × *g,* for 10 min and resuspended in Zymolyase buffer (20 mM K_2_HPO_4_-HCl pH 7.4, 1.2 M sorbitol). 1 mg ZymolyaseF100T (MP Biomedicals, LLC) was added per gram wet weight and the solution was shaken slowly at 30°C for 60 min. This was followed by centrifugation at 4,000 × *g,* for 15 min. The pellet was further resuspended in Zymolyase buffer and centrifuged for a further 15 min at 4,000 × *g.* The pellet was then resuspended in homogenization buffer (20 mM HEPES-KOH pH 7.45, 0.6 M sorbitol, 1 mM EDTA) and lysed with 15 strokes in a glass homogenizer. To separate the cell debris and nuclei from mitochondria the solution was centrifuged at 2000 × *g,* for 20 min and the supernatant collected, followed by further centrifugation at 4500 × *g,* for 20 min. Crude mitochondria were pelleted at centrifugation at of 13,000 × *g,* for 25 min, and further purified on a sucrose gradient in SEM buffer (250 mM sucrose, 20 mM HEPES-KOH pH 7.5, 1 mM EDTA) by ultracentrifugation at 141,000 x *g* for 1 hour. Mitochondrial samples were pooled and lysed in a buffer of 25 mM HEPES-KOH pH 7.5, 100 mM KCl, 25 mM Mg(OAc)_2_, 1.7% Triton X-100, 2 mM DTT). Upon centrifugation at 30,000 × *g,* for 20 min, the supernatant was loaded on a 1 M sucrose cushion in a buffer containing 20 mM HEPES-KOH pH 7.5, 100 mM KCl, 20 mM Mg(OAc)_2_, 1% Triton X-100, 2 mM DTT. The pellet was resuspended in 10 mM Tris-HCl, pH 7.0, 60 mM KCl, 60 mM NH_4_Cl, 10 mM Mg(OAc)_2_, and applied on a 15–40% sucrose gradient prepared in the same buffer. The fractions containing the SSU were collected, sucrose removed and buffer exchanged to 20 mM HEPES-KOH pH 7.5, 50 mM KCl, 5.0 mM Mg(OAc)_2_, 2.0 mM DTT and 0.05% n-dodecyl-β-D-maltoside by passing through a concentrator (Vivaspin) with a 30 kDa molecular weight cutoff. The final concentration of the mitoribosomes measured at an optical density at 280 nm (OD280) was 3.6

#### Production of mtIF3

The sequence of mtIF3/AIM23 gene of yeast *S. cerevisiae* was inserted in pET30a vector between Nde I and Xho I restriction sites. The plasmid was used to transform *E. coli* Rosetta strain. The culture was inoculated by diluting 100 x with LB and grown until OD600 reached the value of 0.8. Protein production was induced by 0.25 mM IPTG at 30 °C for 3 hours. Biomass was collected by centrifugation at 3500x *g* for 5 min and resuspended in native lysis buffer (25 mM NaHPO4, 500 mM NaCl, 25 mM imidazol, pH 7.4). The suspension was sonicated 6 x 10 sec with 20 sec breaks inbetween. The debris pelleted by centrifugation at 30000x *g* for 20 min. The mtIF3 was then purified on HisTrap HP column by metallo-chelating chromatography. The second step of purification and buffer exchange was done on Superdex 75 10/300 GL column in 20 mM HEPES-KOH, 200 mM NaCl, pH 7.0. The fractions were analysed by SDS-PAGE, pooled and concentrated on Vivaspin20 concentrators to 85 µM. Purified mtIF3 was added to the SSU in 5x molar excess and incubated for 30 min.

#### Cryo-EM data collection and image processing

For cryo-EM, 3 μL of ~100 nM (A_260_ 4.0 or 6.0) mitoribosome sample was applied onto a glow-discharged (20 mA for 30 sec) holey-carbon grid (Quantifoil R2/1 copper, mesh 300) coated with continuous carbon (of ~3 nm thickness) and incubated for 30 sec in a controlled environment of 100% humidity and 4ºC temperature. The grids were blotted for 3.5 sec, followed by plunge-freezing in liquid ethane, using a Vitrobot MKIV (FEI/Thermofischer). The datasets were collected on FEI Titan Krios (FEI/Thermofischer) transmission electron microscope operated at 300 keV with a slit width of 20 eV on a GIF quantum energy filter (Gatan). A K2 Summit detector (Gatan) was used at a pixel size of 0.83 Å (magnification of 165,000x) with a dose of 32 electrons/Å^2^ fractionated over 20 frames. A defocus range of 1.2 to 2.8 μm was applied.

Beam-induced motion correction was performed for all data sets using RELION 3.0^103^. Motion-corrected micrographs were used for contrast transfer function (CTF) estimation with GCTF^104^. Particles were picked by Gautomatch (https://www.mrc-lmb.cam.ac.uk/kzhang) with reference-free, followed by reference-aided particle picking procedures. Reference-free 2D classification was carried out to sort useful particles from falsely picked objects, which were then subjected to 3D refinement, followed by a 3D classification with local-angular search. UCFS Chimera^105^ was used to visualize and interpret the maps. 3D classes corresponding to unaligned or low-quality particles were removed. Well-resolved classes were pooled and subjected to 3D refinement and CTF refinement (beam-tilt, per-particle defocus, per-micrograph astigmatism) by RELION 3.1^106^, followed by Bayesian polishing. Particles were separated into multi optics groups based on acquisition areas and date of data collection. Second round of 3D refinement and CTF refinement (beam-tilt, trefoil and fourth-order aberrations, magnification anisotropy, per-particle defocus, per-micrograph astigmatism) were performed, followed by 3D refinement.

To classify the factor binding states, a non-align focus 3D classifications with particle-signal subtraction using the mask covering the factor binding were done with RELION 3.1. The particles of each state were pooled, subtracted signal was reverted and 3D refinement was done with the corresponding solvent mask. To improve the local resolution, the several local masks were prepared and used for local-masked 3D refinements. Nominal resolutions are based on gold-standard, applying the 0.143 criterion on the Fourier Shell Correlation (FSC) between reconstructed half-maps. Maps were subjected to B-factor sharpening and local-resolution filtering by RELION 3.1, superposed to the overall map and combined for the model refinement.

#### Model building, refinement, and analysis

The starting models for SSU was PDB ID 5MRC^74^. These SSU model was rigid body fitted into the maps, followed by manual revision. Initial models of METTL17 and mtIF3 were generated by SWISS-MODEL ^107^. Ligands and specific extensions/insertions were built manually based on the density. *Coot* 0.9 with Ramachandran and torsion restraints ^108^ was used for manual fitting and revision of the model. Geometrical restraints of modified residues and ligands were calculated by Grade Web Server (http://grade.globalphasing.org). Final models were subjected to refinement of energy minimization and ADP estimation by Phenix.real_space_refine^109^ with rotamer restraints without Ramachandran restrains, against the composed maps with B-factor sharpening and local-resolution filtering. Reference restrains was also applied for non-modified protein residues, using the input models from *Coot* as the reference. Metal-coordination restrains were generated by ReadySet in the PHENIX suite and used for the refinement with some modifications. Refined models were validated with MolProbity^110^ and EMRinger^111^ in the PHENIX suite. UCSF ChimeraX 0.91^112^ was used to make figures.

### Quantification and statistical analysis

Experiments were not randomized, and no blinding was used during the data analyses. Data are reported as mean±SD. Statistical analyses were performed using GraphPad Prism 9 .0 and 10.0 software. Unless otherwise stated in the figure legend, two-way ANOVA with Bonferroni’s post-test was used for multiple comparison.

## Supplementary Material

1

2

3

## Figures and Tables

**Table:** KEY RESOURCES TABLE

REAGENT or RESOURCE	SOURCE	IDENTIFIER
Antibodies		
ACTIN	Millipore Sigma	A3853
CDK5RAP1	Proteintech	14740-1-AP
DYKDDDDK TAG	Cell Signaling	2368
FLAG	Millipore Sigma	F1804
FXN	Abcam	ab175402
HSP60	Abcam	ab45134
ISCU	Santa Cruz	sc-373694
LIAS	Proteintech	11577-1-AP
LYRM4	Aviva Systems Biology	ARP57407_P050
METTL17	Atlas	HPA002955
NFS1	Santa Cruz	sc-365308
OXPHOS	Abcam	ab110411
POLD1	Proteintech	15646-1-AP
TUBULIN	Thermo Fisher	MA5-16308
TUBULIN	Cell Signaling	2128
IRDye 800CW Goat anti-Mouse IgG (H + L)	LI-COR Biosciences	926-32210
IRDye 800CW Goat anti-Rabbit IgG (H + L)	LI-COR Biosciences	926-32211
IRDye 680RD Goat anti-Mouse IgG (H + L)	LI-COR Biosciences	926-68070
IRDye 680RD Goat anti-Rabbit IgG (H + L)	LI-COR Biosciences	926-68071
Bacterial and virus strains		
OverExpress C41(DE3)	Millipore Sigma	CMC0017
Chemicals, peptides, and recombinant proteins		
DMEM	GIBCO	11995073
DMEM, no glucose	GIBCO	11966025
DMEM, high glucose, no glutamine, no methionine, no cystine	GIBCO	21013024
Seahorse XF Base Medium with 5 mM HEPES	Agilent	103575-100
Sodium Pyruvate	GIBCO	11360070
L-Glutamine	GIBCO	25030081
Fetal Bovine Serum	GIBCO	26140079
Fetal Bovine Serum, dialyzed	GIBCO	26400044
Galactose	Millipore Sigma	PHR1206
Puromycin Dihydrochloride	GIBCO	A1113803
Geneticin (G418 Sulfate)	GIBCO	10131035
Emetine	Millipore Sigma	E2375
Chloramphenicol	Millipore Sigma	C0378
Oligomycin A	Millipore Sigma	75351
Bam15	Millipore Sigma	SML1760
Antimycin A from *Streptomyces* sp.	Millipore Sigma	A8674
Piericidin A	Santa Cruz	Sc-202287
EXPRESS [35S]-protein labeling mix	PerkinElmer	NEG772
SEA BLOCK Blocking Buffer	Thermo Fisher Scientific	37527
Critical commercial assays		
Lipofectamine 2000	Thermo Fisher Scientific	11668019
Pierce 660nm Protein Assay Kit	Thermo Fisher Scientific	22662
BCA protein assay kit	Thermo Fisher Scientific	23227
Novex 4-20% Tris-Glycine Mini Gels	Thermo Fisher Scientific	XP04202BOX
Trans-Blot Turbo Midi Nitrocellulose Transfer Packs	BioRad	1704159
RNeasy Mini Kit	Qiagen	74106
M-MLV Reverse Transcriptase	Promega	M1701
TaqMan Gene Expression Master Mix	Thermo Fisher Scientific	4369016
TaqMan *12S*	Thermo Fisher Scientific	Hs02596859_g1
TaqMan *16S*	Thermo Fisher Scientific	Hs02596860_s1
TaqMan *METTL17*	Thermo Fisher Scientific	Hs00224159_m1
TaqMan *TBP*	Thermo Fisher Scientific	Hs00427620_m1
Seahorse XFe96 FluxPaks	Agilent	102416-100
Deposited data		
Proteomic data for control and FXN depleted cells	This paper	PRIDE: PXD045443
Sequencing data from FXN genetic interaction screen	This paper	GEO: GSE242192
Structure of the small subunit of yeast mitochondrial ribosome in complex with METTL17/Rsm22	This paper	PDB: 8OM2
Structure of the small subunit of yeast mitochondrial ribosome in complex with IF3/Aim23	This paper	PDB: 8OM3
Structure of the small subunit of yeast mitochondrial ribosome	This paper	PDB: 8OM4
Experimental models: Cell lines		
K562	ATCC	CCL-243
293T	ATCC	CRL-3216
A549	ATCC	CCL-185
Software and algorithms		
R	The R Foundation	http://www.R-project.org
Prism v.9, v.10	GraphPad Software	https://www.graphpad.com/scientific-software/prism/
Excel for Microsoft 365, v2208	Microsoft	https://www.microsoft.com/
Seahorse Wave Desktop Software	Agilent	https://www.agilent.com/en/products/cell-analysis/cell-analysis-software/data-analysis/wave-desktop-2-6
Gene set enrichment analysis (GSEA)	The Broad Institute	http://software.broadinstitute.org/gsea/index.jsp
UCSF ChimeraX 0.91	UCSF Resource for Biocomputing, Visualization, and Informatics, UC San Francisco	https://www.cgl.ucsf.edu/chimerax/download.html
Cancer Dependency Map	Project Achilles, The Broad Institute	https://depmap.org/portal/
BioPlex 3.0	Gygi Lab, Harvard Medical School	https://bioplex.hms.harvard.edu/

## Highlights:

- The Fe-S proteome is destabilized when the synthesis of its cofactor is impaired
- METTL17, a mitoribosome assembly factor, is depleted without Fe-S cluster biosynthesis
- METTL17 binds a previously unrecognized [4Fe4S]^2+^ cluster
- Overexpression of METTL17 rescues the bioenergetic defects of frataxin null cells
