# Supplementary material for: METTL17 is an Fe-S cluster checkpoint for mitochondrial translation
Source: Mol Cell. Author manuscript; Available in PMC 2024 Apr 26. (PMC11046306; doi:10.1016/j.molcel.2023.12.016)
Supplement: 1 [file NIHMS1958834-supplement-1.pdf]

METTL17 is an Fe-S cluster checkpoint for mitochondrial translation

SUPPLEMENTAL FIGURES AND LEGENDS

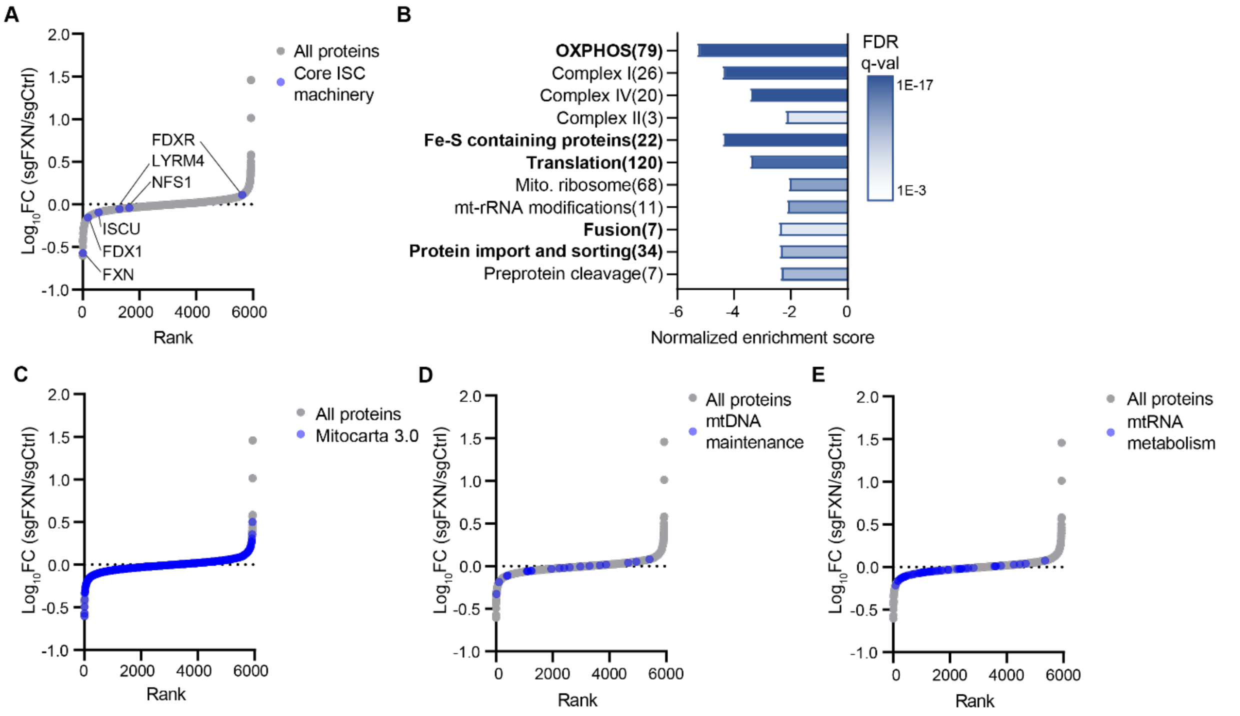

Fig S1: OXPHOS, but not ISC machinery or the mito-proteome, is depleted in the absence of FXN. Related to Figure 1.

A. Waterfall plots of protein fold change in FXN/Control cells, highlighting proteins in the core ISC machinery. B. Analysis of MitoPathways depleted in FXN edited cells. Pathways in bold are independent. In parentheses are the number of proteins enriched in each term. C-E. Waterfall plots of protein fold change in FXN/Control cells, highlighting proteins in MitoCarta 3.0 (C) mtDNA maintenance proteins (D) and mtRNA metabolism (E).

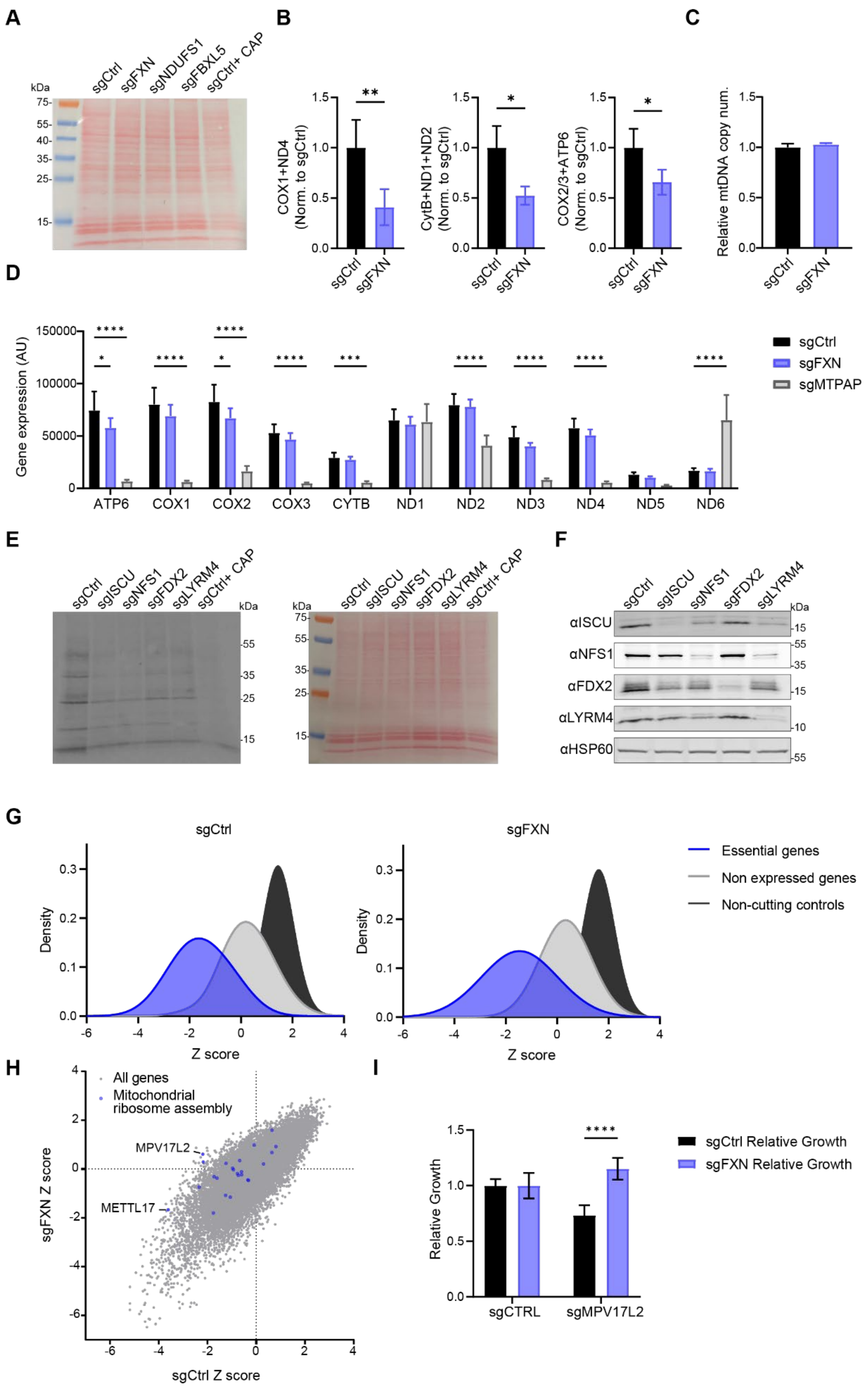
A
sgCtrl
sgFXN
sgNDUFS1
sgFBXL5
sgCtrl+ CAP
kDa
B
COX1+ND4 (Norm. to sgCtrl)
CytB+ND1+ND2 (Norm. to sgCtrl)
COX2/3+ATP6 (Norm. to sgCtrl)
C
Relative mtDNA copy num.
D
Gene expression (AU)
sgCtrl
sgFXN
sgMTPAP
ATP6
COX1
COX2
COX3
CYTB
ND1
ND2
ND3
ND4
ND5
ND6
E
sgISCU
sgNFS1
sgFDX2
sgLYRM4
F
αISCU
αNFS1
αFDX2
αLYRM4
αHSP60
G
Density
Z score
Essential genes
Non expressed genes
Non-cutting controls
H
All genes
Mitochondrial ribosome assembly
MPV17L2
METTL17
sgFXN Z score
sgCtrl Z score
I
Relative Growth
sgCTRL
sgMPV17L2
sgCtrl Relative Growth
sgFXN Relative Growth

Figs S2: mtDNA replication and transcription is not significantly altered in FXN depleted cells. Related to Figure 2.

A. Ponceau S staining of the protein membrane found in Fig2A. B. Quantification of nascent mtDNA translation products in sgFXN cells, normalized to sgCtrl (n=3 for all samples). C. qPCR for mtDNA copy number in sgCtrl and sgFXN cells (n=6 for both samples). D. Mitostring assay examining the levels of mtDNA encoded transcripts in sgCtrl, sgFXN and sgMTPAP cells (n=4 for all samples) E. Left- Mitochondrial translation, as assessed by autoradiography after 35S-methionine/cysteine labeling, of cells edited for ISCU, NFS1, FDX2 and LYRM4. All cells were treated with 200µg/mL emetine, and control cells in the last lane were also treated with 50µg/µL chloramphenicol. Right- Ponceau S staining of the protein membrane. F. Immunoblots examining ISCU, NFS1, FDX2, LYRM4 or the loading control HSP60 in cells edited for ISCU, NFS1, FDX2 and LYRM4. G. Histograms of the Z score of cutting controls, non-expressed genes and essential genes as defined previously[113] in the genetic interaction screens performed in sgCtrl or sgFXN cells. H. Scatterplot of Z scores showing knockouts growth in sgCtrl vs. sgFXN backgrounds. All the mitochondrial ribosome assembly genes, as defined by MitoCarta 3.0, are highlighted in blue. I. Relative growth rates of cells edited for control or MPV17L2 gene, on the background on sgCtrl or sgFXN. Growth rates were normalized to the unedited growth rate for each strain (n=6 for all samples). All bar plots show mean ± SD. *=$p < 0.05$, **=$p < 0.01$, ***=$p < 0.001$, ****=$p < 0.0001$. Two-way ANOVA with Bonferroni's post-test.

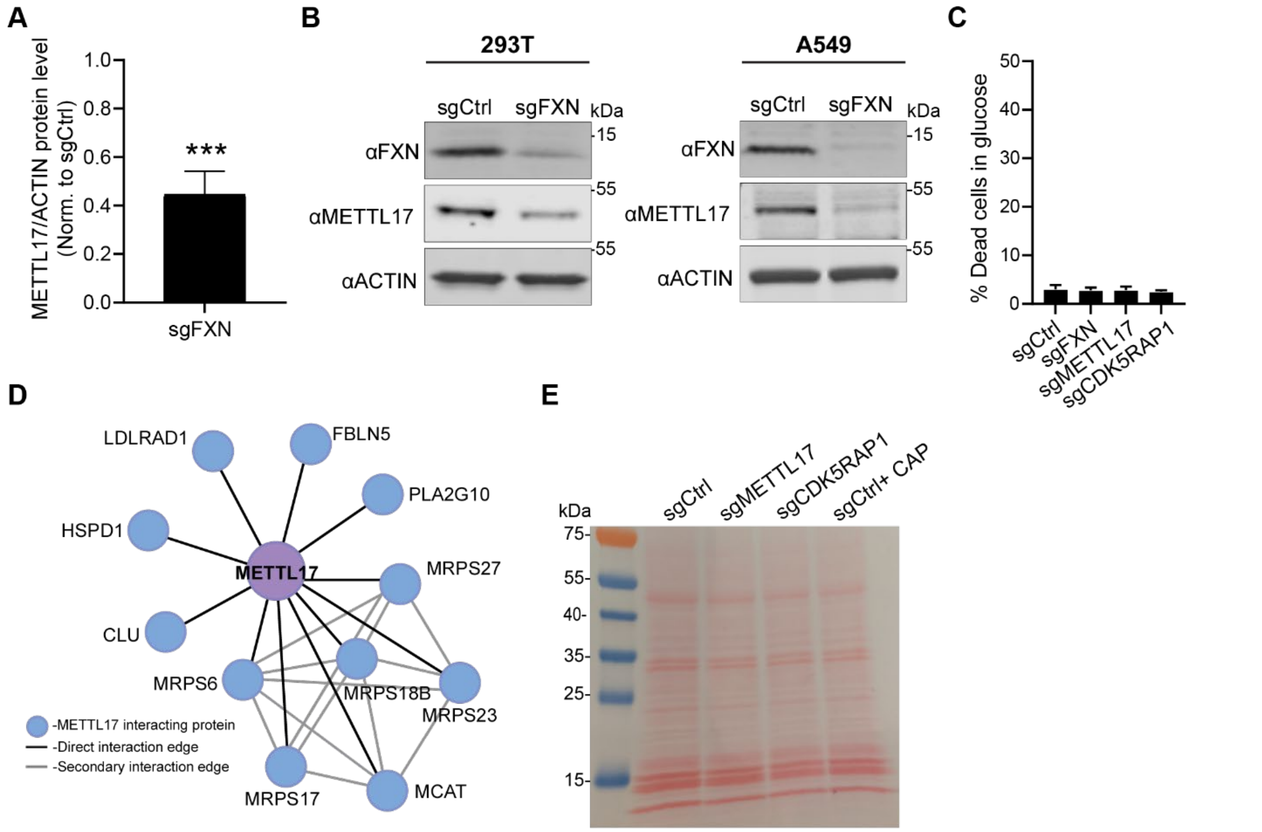

Fig S3: METTL17 is depleted in FXN edited cells and is linked to mitochondrial translation. Related to Figure 3.

A. Quantification of METTL17/ACTIN protein levels from sgFXN K562 cells, normalized to sgCtrl edited cells (n=3) B. Immunoblot for FXN, METTL17 and the loading control actin in 293T and A549 cells edited with control or FXN guides. C. Cells edited for control, FXN, METTL17 and CDK5RAP1 genes were grown for 24h in glucose media, and viability was assessed on each background (n=3 for all samples). D. Protein-protein interactions identified for METTL17 in 293T cells[58] E. Ponceau S staining of the protein membrane found in Fig3E. All bar plots show mean ± SD. ***=p < 0.001, Two-tailed t test.

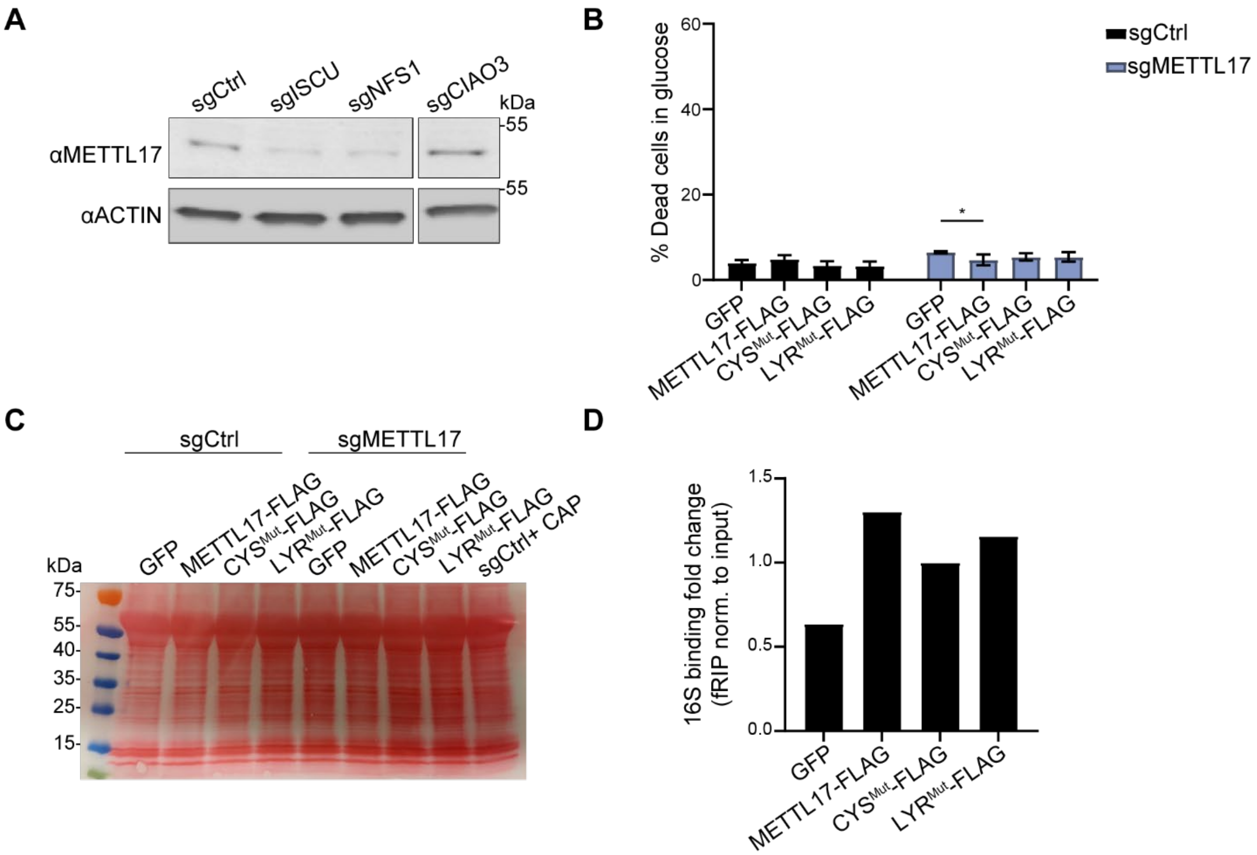

Fig S4: METTL17 has characteristics of an Fe-S cluster binding protein. Related to Figure 4.

A. Immunoblot for METTL17 and the loading control actin in cells edited for control, ISC genes (ISCU and NFS1) or a CIA gene (CIAO3). B. Cells edited for control or METTL17 genes and expressing WT or mutant forms of METTL17 were grown for 24h in glucose media, and viability was assessed on each background (n=4 for all samples). C. Ponceau S staining of the protein membrane found in Fig4E. D. Formaldehyde-linked RNA immunoprecipitation of the 16S to GFP, METTL17-FLAG, CYSMut-FLAG or LYRMut-FLAG proteins. Results were normalized to input construct and 16S levels (n=2 for all samples). Bar plot in fig S4B shows mean ± SD. *=p < 0.05, Two-way ANOVA with Bonferroni's post-test.

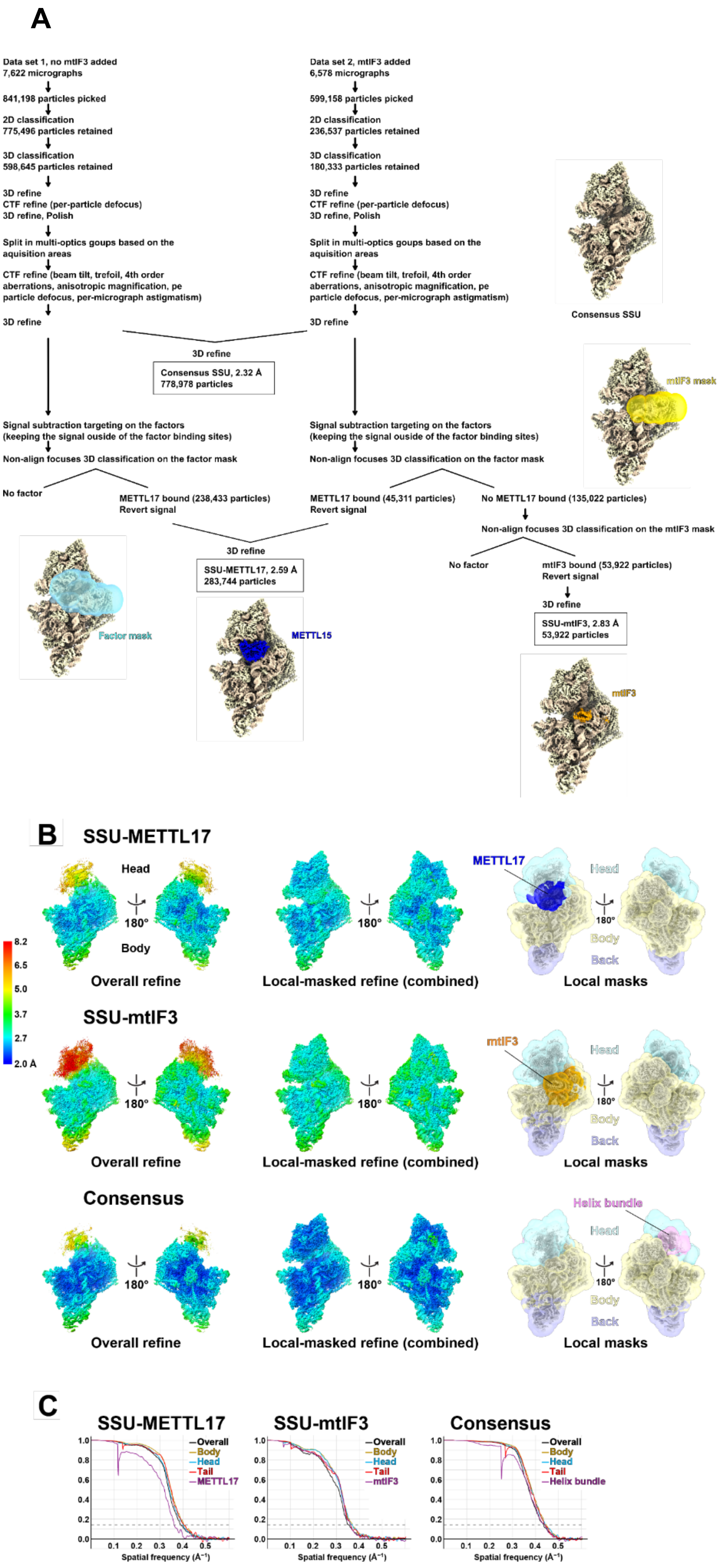
A
Data set 1, no mtIF3 added
7,622 micrographs
841,198 particles picked
2D classification
775,496 particles retained
3D classification
598,645 particles retained
3D refine
CTF refine (per-particle defocus)
3D refine, Polish
Split in multi-optics goups based on the aquisition areas
CTF refine (beam tilt, trefoil, 4th order aberrations, anisotropic magnification, pe particle defocus, per-micrograph astigmatism)
3D refine
Data set 2, mtIF3 added
6,578 micrographs
599,158 particles picked
2D classification
236,537 particles retained
3D classification
180,333 particles retained
3D refine
CTF refine (per-particle defocus)
3D refine, Polish
Split in multi-optics goups based on the aquisition areas
CTF refine (beam tilt, trefoil, 4th order aberrations, anisotropic magnification, pe particle defocus, per-micrograph astigmatism)
3D refine
Consensus SSU
3D refine
Consensus SSU, 2.32 Å
778,978 particles
mtIF3 mask
Signal subtraction targeting on the factors (keeping the signal ouside of the factor binding sites)
Non-align focuses 3D classification on the factor mask
No factor
METTL17 bound (238,433 particles)
Revert signal
METTL17 bound (45,311 particles)
Revert signal
No METTL17 bound (135,022 particles)
Non-align focuses 3D classification on the mtIF3 mask
3D refine
SSU-METTL17, 2.59 Å
283,744 particles
No factor
mtIF3 bound (53,922 particles)
Revert signal
3D refine
SSU-mtIF3, 2.83 Å
53,922 particles
Factor mask
METTL15
mtIF3
B
SSU-METTL17
Head
Body
180°
8.2
6.5
5.0
3.7
2.7
2.0 Å
Overall refine
Local-masked refine (combined)
METTL17
Head
Body
Back
Local masks
SSU-mtIF3
mtIF3
Consensus
Helix bundle
C
SSU-METTL17
SSU-mtIF3
Consensus
Overall
Body
Head
Tail
METTL17
mtIF3
Helix bundle
Spatial frequency (Å⁻¹)

Fig S5: Cryo-EM image processing. Related to Figure 6.

A. The data processing scheme. B. Overall maps, combined maps of the local-masked refinements colored by local resolution are shown for SSU-METTL17 (top), SSU-mtIF3 (middle), and SSU (bottom). C. Fourier Shell Correlation curves of the half maps and local-masked refinements. The 0.143 criterion is shown as dashed lines.

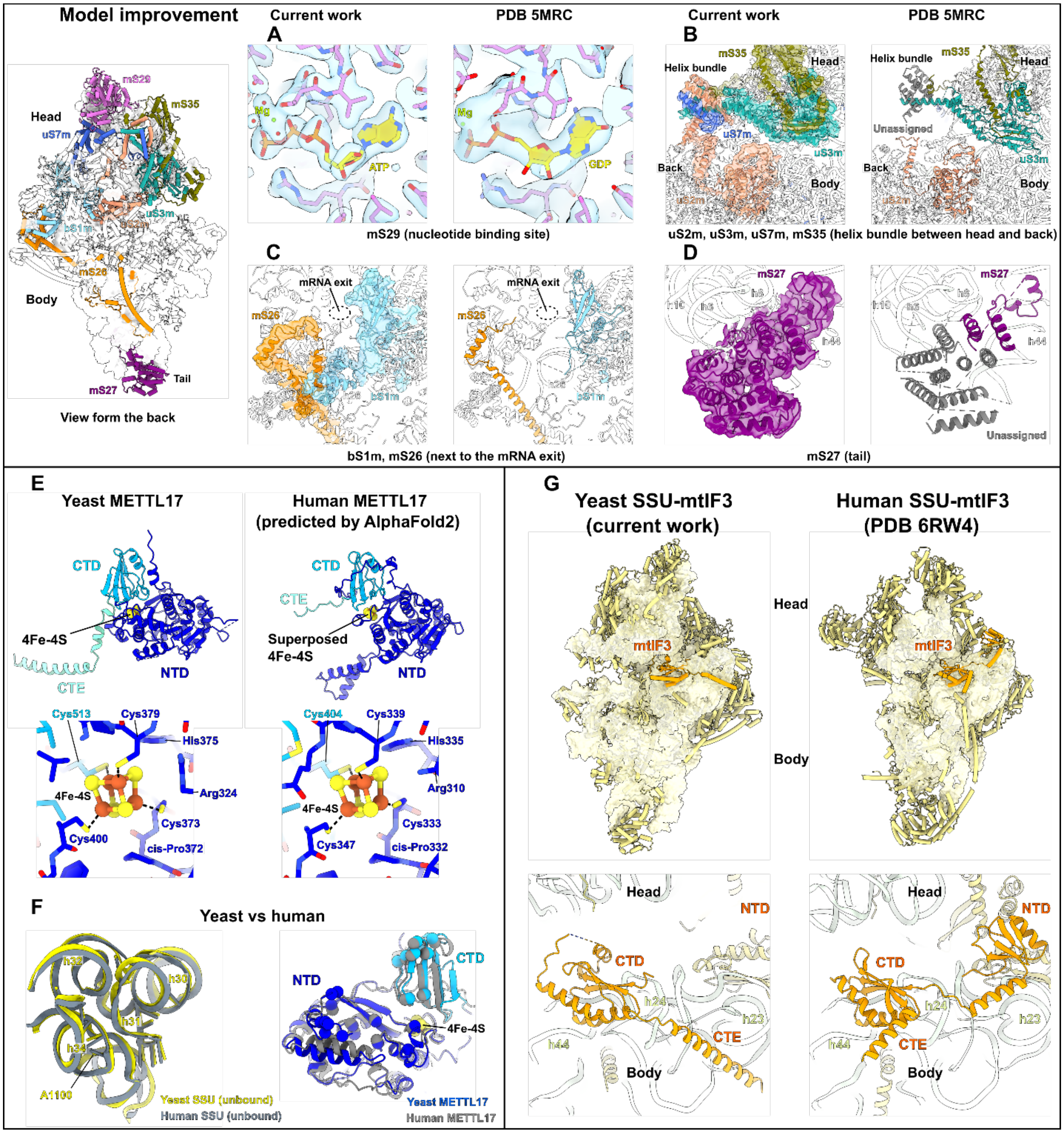

Fig S6: Cryo-EM structure of the yeast SSU, METTL17 and mtIF3. Related to Figure 6.

A-D. Improvements in the model of *S. cerevisiae* mitoribosome. Overview of the SSU model from the back with improved proteins colored. The close-up views show modeled elements with their corresponding density map, and equivalent regions from previous studies[74] are shown for comparison. A. The nucleotide density for mS29 in the SSU head. B. The density and corresponding models of uS2m, uS3m, uS7m, mS35 that form a previously unsigned helix bundle between the head and body. C. Complete models for bS1m and mS26 that form contacts at the mRNA channel exit. D. Remodeled and reannotated mS27 interacts with h44, which was previously partially built as poly-Ala and named mS44. E. Comparison between the yeast cryo-EM model and human *AlphaFold2*[65] prediction of METTL17 shows that the

predicted conformations of the NTD (blue) and CTD (light blue) are highly similar, including the coordination of the 4Fe-4S shown in the close-up view, and structural differences are observed only in the terminal extensions. The Fe-S cluster in the human model was placed by superposing that of the yeast cryo-EM structure. F. Comparison between yeast and human SSU (left) and METTL17 (right) interfaces. Phylum-specific protein extensions have been removed for clarity. The residues involved in interactions are shown in sticks for RNA and spheres for protein. G. Comparison between yeast and human[77] SSU-IF3 complex with close-up views showing that the binding of the mtIF3 CTD (orange) is conserved. Thus, human mtIF3 has similar structural characteristics and would also clash with METTL17 on the SSU. The NTD of mtIF3 is not resolved in our map, and thus hasn't been modelled. On the other hand, the C-terminal extensions (CTE) forming a helix have different orientations. The CTE in yeast keeps contacting the rRNA in the body, whereas that of human is exposed.

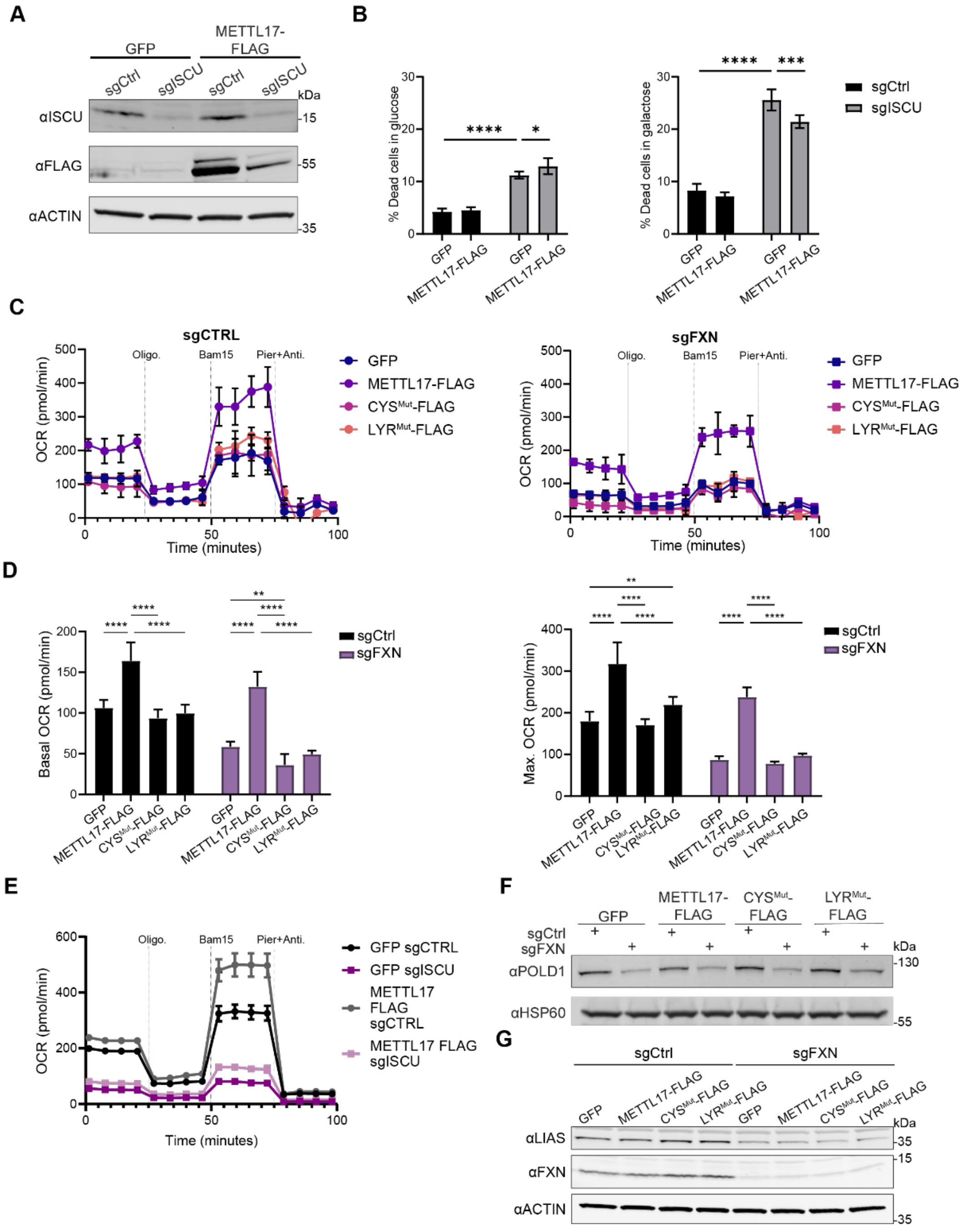

Fig S7: METTL17 overexpression restores the faulty mitochondrial bioenergetics of FXN depleted cells. Related to Figure 7.

A. Immunoblot of ISCU, FLAG tagged METTL17 construct or the loading control HSP60 on cells edited with control or ISCU guides. B. Control or ISCU edited cells expressing GFP or METTL17-FLAG constructs were grown for 24h in glucose (left) or galactose (right), following which their viability was assessed (n=6 for all samples) C. Oxygen consumption rate of Control (top) or FXN (bottom) edited cells expressing GFP, METTL17-FLAG, CYSMut-FLAG or LYRMut-FLAG. Cells were sequentially treated

with oligomycin, Bam15 and Piericidin A+ Antimycin A. D. Basal (left) and maximal (right) OCR of Control or FXN edited cells expressing GFP or METTL17-FLAG (n=7 for all samples). F. Immunoblots examining POLD1 or the loading control HSP60 in Control or FXN edited cells expressing GFP, METTL17-FLAG, CYS^{Mut}-FLAG or LYR^{Mut}-FLAG constructs. G. Immunoblots examining LIAS, FXN or the loading control ACTIN in Control or FXN edited cells expressing GFP, METTL17-FLAG, CYS^{Mut}-FLAG or LYR^{Mut}-FLAG constructs. All bar plots show mean ± SD. **=$p < 0.01$, ****=$p < 0.0001$. Two-way ANOVA with Bonferroni's post-test.

	SSU-METTL17 (PDB ID: 8OM2) (EMD-16966)	SSU-mtIF3 (PDB ID: 8OM3) (EMD-16967)	Consensus SSU (PDB ID: 8OM4) (EMD-16968)
**Data collection and Processing**			
Microscope	Titan Krios	Titan Krios	Titan Krios
Voltage (kV)	300	300	300
Camera	K2 Summit	K2 Summit	K2 Summit
Magnification	165,000	165,000	165,000
Pixel size at detector (Å/pixel)	0.83	0.83	0.83
Total electron exposure (e^{-}/Å^2)	30–32	30	30–32
Exposure rate (e^{-}/pixel/sec)	5.6–5.9	5.9	5.6–5.9
Number of frames	20	20	20
Defocus range (μm)	−1.2 to −2.8	−1.2 to −2.6	−1.2 to −2.8
Energy filter slit width (V)	20	20	20
Micrographs collected (no.)	14,200	6,578	14,200
Final particles (no.)	283,744	53,922	778,978
Point group	C_1	C_1	C_1
Resolution (global, Å) FSC 0.143 (masked) (Overall/ body/ head/ tail/ factor)	2.57/ 2.47/ 2.51/ 2.49/ -/ 2.77	2.87/ 2.71/ 2.71/ 2.77/ -/ -	2.32/ 2.25/ 2.28/ 2.28/ 2.29/ -
Map-sharpening B factor (Å^2) (Overall/ body/ head/ tail/ helix bundle/ METTL17)	−41/ −41/ −40/ −50/ -/ −67	−37/ −34/ −37/ −46/ -/ -	−43/ −43/ −42/ −53/ −55/ -
EMDB deposition of constituent maps (Overall/ body/ head/ tail/ helix bundle/ METTL17)	EMD-17089/ EMD-17090/ EMD-17091/ EMD-17092/ -/ EMD-17093	EMD-17094/ EMD-17095/ EMD-17096/ EMD-17097/ -/ -	EMD-17098/ EMD-17099/ EMD-17100/ EMD-17101/ EMD-17102/ -
**Model composition**			
Atoms (non-hydrogen/ hydrogen)	95,205/ 77,625	91,388/ 75,332	92,918/ 74,078
Chains (RNA/ protein)	1/ 34	1/ 34	1/ 33
RNA residues (non-modified/ modified)	1,488/ 0	1,488/ 0	1,488/ 0
Protein residues (non-modified/ modified)	7478/ 1 (*N*-acetyl Ser)	7,192/ 1 (*N*-acetyl Ser)	7,048/ 1 (*N*-acetyl Ser)
Metal ions (Mg^{2+}/ K^{+})	90/ 35	96/ 26	89/ 37
Ligands (ATP/ 4Fe-4S/ β-DDM)	1/ 1/ 1	1/ 0/ 1	1/ 0/ 1
Waters	3,014	1,484	4,206
**Model Refinement**			
Model-Map CC (mask/ box/ peaks/ volume)	0.83/ 0.87/ 0.80/ 0.84	0.83/ 0.88/ 0.80/ 0.84	0.84/ 0.88/ 0.82/ 0.85
Resolution (Å) by model-to-map FSC, threshold 0.50 (masked/ unmasked)	2.18/ 2.19	2.41/ 2.41	2.03/ 2.03
Average B factor (Å^2) (RNA/ protein/ metal ion or ligand/ water)	41/ 42/ 31/ 27	48/ 49/ 32/ 28	36/ 35/ 25/ 24
R.m.s. deviations, bond lengths (Å)/ bond angles (°)	0.002/ 0.427	0.002/ 0.425	0.002/ 0.436
**Validation**			
MolProbity score	1.01	0.95	0.97
CaBLAM outliers (%)	0.92	0.84	0.84
Clash score	1.90	1.70	1.71
Rotamer outliers (%)	0.06	0.03	0.03
C_β deviations	0	0	0
EMRinger score	5.05	4.61	5.79
Ramachandran plot (%) (Favored/ allowed/ disallowed)	97.74/ 2.26/ 0.00	97.89/ 2.10 / 0.01	97.81/ 2.19/ 0.00

Table S1. Data collection, processing, model refinement and validation statistics. Related to Figure 6.
